# GRAS transcription factors regulate cell division planes in moss overriding the default rule

**DOI:** 10.1073/pnas.2210632120

**Published:** 2023-01-20

**Authors:** Masaki Ishikawa, Ayaka Fujiwara, Ken Kosetsu, Yuta Horiuchi, Naoya Kamamoto, Naoyuki Umakawa, Yosuke Tamada, Liechi Zhang, Katsuyoshi Matsushita, Gergo Palfalvi, Tomoaki Nishiyama, Sota Kitasaki, Yuri Masuda, Yoshiki Shiroza, Munenori Kitagawa, Toru Nakamura, Hongchang Cui, Yuji Hiwatashi, Yukiko Kabeya, Shuji Shigenobu, Tsuyoshi Aoyama, Kagayaki Kato, Takashi Murata, Koichi Fujimoto, Philip N. Benfey, Mitsuyasu Hasebe, Rumiko Kofuji

**Affiliations:** ^a^Division of Evolutionary Biology, National Institute for Basic Biology, Okazaki 444-8585, Japan; ^b^Department of Basic Biology, The Graduate School for Advanced Studies (SOKENDAI), Okazaki 444-8585, Japan; ^c^Graduate School of Natural Science and Technology, Kanazawa University, Kanazawa 920-1192, Japan; ^d^Department of Biological Sciences, Graduate School of Science, Osaka University, Toyonaka 560-0043, Japan; ^e^School of Engineering, Utsunomiya University, Utsunomiya 321-8585, Japan; ^f^Division of Integrated Omics Research, Research Center for Experimental Modeling of Human Disease, Kanazawa University, Kanazawa 920-0934, Japan; ^g^Department of Biology, Kanazawa University, Kanazawa 920-1192, Japan; ^h^Department of Biology, Duke University, Durham, NC 27516; ^i^HHMI, Duke University, Durham, NC 27516; ^j^Department of Biological Science, Florida State University, Tallahassee, FL 32306-4295; ^k^School of Food Industrial Sciences, Miyagi University, Sendai 982-0215, Japan; ^l^Bioimage Informatics Group, Exploratory Research Center on Life and Living Systems (ExCELLS), National Institutes of Natural Sciences, Okazaki 444-8585, Japan; ^m^Interdisciplinary Research Unit, National Institute for Basic Biology, Okazaki 444-8585, Japan; ^n^Department of Applied Bioscience, Kanagawa Institute of Technology, Atsugi 243-0292, Japan; ^o^Faculty of Biological Science and Technology, Institute of Science and Engineering, Kanazawa University, Kanazawa 920-1192, Japan

**Keywords:** GRAS TFs, geometry rule, cell division orientation, horizontal gene transfer

## Abstract

Plant cells are surrounded by a cell wall and do not migrate, which makes the regulation of cell division orientation crucial for development. Regulatory mechanisms controlling cell division orientation may have contributed to the evolution of body organization in land plants. The GRAS family of transcription factors was transferred horizontally from soil bacteria to an algal common ancestor of land plants. *SHORTROOT* (*SHR*) and *SCARECROW* (*SCR*) genes in this family regulate formative periclinal cell divisions in the roots of flowering plants, but their roles in nonflowering plants and their evolution have not been studied in relation to body organization. Here, we show that *SHR* cell autonomously inhibits formative periclinal cell divisions indispensable for leaf vein formation in the moss *Physcomitrium patens*, and SHR expression is positively and negatively regulated by *SCR* and the GRAS member *LATERAL SUPPRESSOR*, respectively. While precursor cells of a leaf vein lacking SHR usually follow the geometry rule of dividing along the division plane with the minimum surface area, SHR overrides this rule and forces cells to divide nonpericlinally. Together, these results imply that these bacterially derived GRAS transcription factors were involved in the establishment of the genetic regulatory networks modulating cell division orientation in the common ancestor of land plants and were later adapted to function in flowering plant and moss lineages for their specific body organizations.

## Significance

Plant cells are surrounded by a cell wall and do not migrate, making the regulation of cell division orientation critical to the establishment of plant body shape. A gene encoding a GRAS transcription factor was transferred from soil bacteria to plants, and its descendent genes now regulate formative cell divisions in flowering plants. Here, we show that three GRAS proteins participate in leaf vein formation by regulating cell division orientation in the moss *Physcomitrium patens*. We propose that the acquisition of GRAS genes contributed to the genetic regulatory networks controlling cell division orientation in the ancestor of land plants, and this gene family then underwent expansion and adaptation in flowering plant and moss lineages to specify body plans.

Evolution of development is caused by the alteration of genetic regulatory networks with spatiotemporal cooption of genes from the ancestral genome, as well as by gene duplications and/or whole-genome duplication (1, 2). Furthermore, horizontal gene transfer, the acquisition of genes from distantly related organisms may result in the evolution of traits (3, 4). Horizontally transferred genes have been reported in several land plants (5) shown to have acquired adaptive functions in some cases, including abscisic acid receptors in the land plant ancestor (6), a fern photoreceptor (7), and a C4 photosynthesis component in grasses (8). In the context of development, the DNA-binding domain present in APETALA2 (AP2)/ETHYLENE RESPONSE FACTOR (ERF) transcription factors is thought to have been transferred from cyanobacteria to the green algae ancestral to land plants (9). AP2/ERF proteins participate in the determination of floral organ identity in angiosperms (10) and the formation of leafy shoots in the mosses (11) as well as in stem cell formation (12–14).

GRAS proteins function in various aspects of land plant development (15) and are inferred to have been acquired by horizontal gene transfer from soil bacteria to the common ancestor of land plants and their sister Zygnematalean green algae (6, 15). The GRAS family member SHORTROOT (SHR), together with another GRAS transcription factor SCARECROW (SCR), governs the formative periclinal cell division of cortex/endodermis initial (CEI) cells in *Arabidopsis thaliana* (Arabidopsis) roots (16, 17). *SHR* is translated in stele cells and moves to CEIs to induce *SCR* transcription (18). SHR and SCR directly regulate the transcription of the cell cycle regulator D-type cyclin *CYCD6;1* during periclinal cell division (19).

The evolution of formative periclinal cell divisions was crucial for establishing the basic body organization of land plants. With the exception of charophytes, which evolved their complex body organization in parallel to land plants, multicellular green algae closely related to land plants take on a filamentous (unidimensional) or planar (two-dimensional) body organization, suggesting that three-dimensional body organization evolved with the common ancestor of land plants, after its divergence from algae (20, 21). Together with the evolution of apical stem cells with rotating division planes (20), the evolution of the formative periclinal cell division machinery to produce outer and inner cells resulted in the differentiation of the epidermis and cortex and the formation of new organs, each composed of outer cell layers and inner differently differentiated cells: archegonia bearing an egg cell, antheridia bearing sperms, sporangia bearing meiotic spores, and leaf veins comprising water-conducting cells. Therefore, the evolution of mechanisms regulating cell division orientation was crucial for the present body organization of land plants.

Two possible default cell division orientation rules have been proposed in land plants (22). According to the first, the division plane aligns to the direction of maximal tensile stress (23–26); while the second rule posits that the minimal surface area is selected in a probabilistic manner (27–29). The first rule is more general, while the second rule is applicable to early embryos (30) and relatively flat tissues experiencing isotropic stress (22, 26, 27). Internal and external cues can override the default rules. Representative internal cues include the regulation of transcription of cell cycle regulators (19, 31, 32), nuclear movement, and protein localization (33), while the most prominent external cues are the phytohormone auxin (34, 35) and small peptides (36, 37). Since the default division rules depend on the cell wall and are applicable to all plant tissues, including green algae with a simple organization, the evolution of internal and external cues to regulate cell division orientation is likely to have contributed to the establishment of the complex body organization seen in land plants. SHR and SCR are potential regulators for these cues in their roles as transcription factors, as they regulate a formative periclinal cell division in angiosperms.

To determine whether SHR and SCR globally regulate formative periclinal cell divisions in land plants, we investigated their functions in the moss *Physcomitrium patens* (Physcomitrium), which occupies a position in the basal lineage of land plants. In this study, we demonstrate that *SHR*, *SCR*, and another GRAS member orthologous to Arabidopsis *LATERAL SUPPRESSOR* (*LAS*) together regulate the cell division orientation with overriding the default geometry rule to form water-conducting tissue, a shared character in land plants. We propose that horizontally transferred GRAS genes contributed to the evolution of body plan in land plants through the regulation of cell division orientation.

## Results

### Physcomitrium SHORTROOT Prevents Periclinal Cell Division in Leaf Cells.

**Fig. 1.**
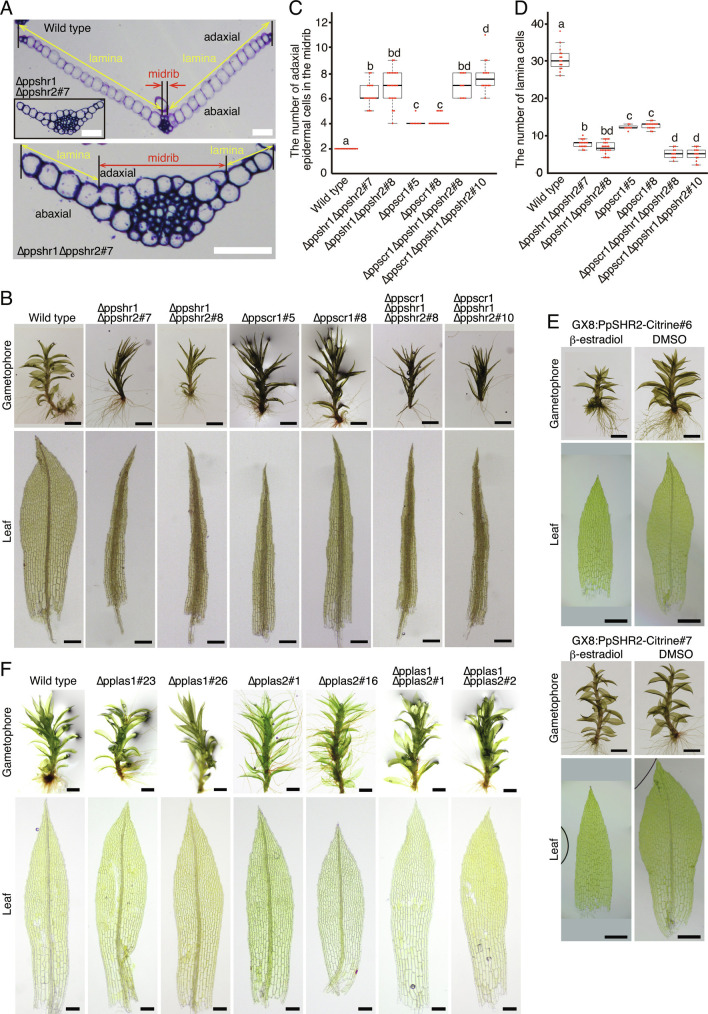
Regulation of leaf development by PpSHR1, PpSHR2, PpSCR1, PpLAS1, and PpLAS2. (*A*) Representative toluidine blue-stained transverse sections of leaves in wild-type and ∆ppshr1∆ppshr2#7 plants. The *Inset* in *Upper* panel is shown at higher magnification in *Lower* panel. Yellow and red arrows indicate the lamina (with a single cell layer) and the midrib (with two or more layers), respectively. (Scale bars, 50 µm.) (*B*) Representative gametophores and mature leaves of wild-type and GRAS gene-deletion mutant plants. (Scale bars, 1 mm in *Upper* panels; 200 µm in *Lower* panels.). (*C* and *D*) The number of adaxial epidermal cells in the midrib (*C*) and the number of lamina cells (*D*). Boxplots represent values between upper and lower quartile, with the median value as black horizontal lines and the error bars the representing SD. Individual data are plotted as red dots (*n* = 8 to 24 leaves). Lowercase letters indicate significant differences (one-way ANOVA and Tukey’s test, *P* < 0.05). Mature leaves in the 8th to 15th positions from the oldest leaves were excised from gametophores. (*E*) Representative gametophores and mature leaves of PpSHR2–Citrine-induced plants. Gametophores were cultivated in liquid BCD medium with β-estradiol (*Left*) or DMSO (*Right*) for 30 d. (Scale bars, 1 mm in *Upper* panels; 100 µm in *Lower* panels.) (*F*) Representative gametophores and mature leaves of wild-type and *PpLAS* deletion mutant plants. (Scale bars, 1 mm in *Upper* panels; 200 µm in *Lower* panels.)

The Physcomitrium genome encodes two genes orthologous to Arabidopsis *SHR*: *Physcomitrium patens SHR1* (*PpSHR1*) and *PpSHR2* (*SI Appendix*, Fig. S1). To investigate their functions, we generated single and double deletion mutant lines (*SI Appendix*, Fig. S2 *A*–*E*). Each Physcomitrium phyllid (hereafter called “leaf”) is composed of the lamina, with a single-cell layer, and the midrib with two or more cell layers (Fig. 1*A*). The widths of the lamina and midrib were narrower and wider, respectively, than those of wild type in some of the single deletion lines (*SI Appendix*, Fig. S2 *F* and *G*) and in all of the double deletion lines (Fig. 1*B*). Furthermore, in transverse sections of the middle of a leaf (Fig. 1*A*), the number of cells in the lamina was decreased while the number of adaxial epidermal cells in the midrib was increased in the double deletion lines as compared with wild type (Fig. 1 *C* and *D*). This indicates that *PpSHR*s regulate cell divisions in leaves. As the observed morphological differences in gametophore leaves may be a result of complex direct and indirect effects during development, we focused on the first cellular difference during leaf development between wild-type and Δppshr1Δppshr2 double deletion plants.

**Fig. 2.**
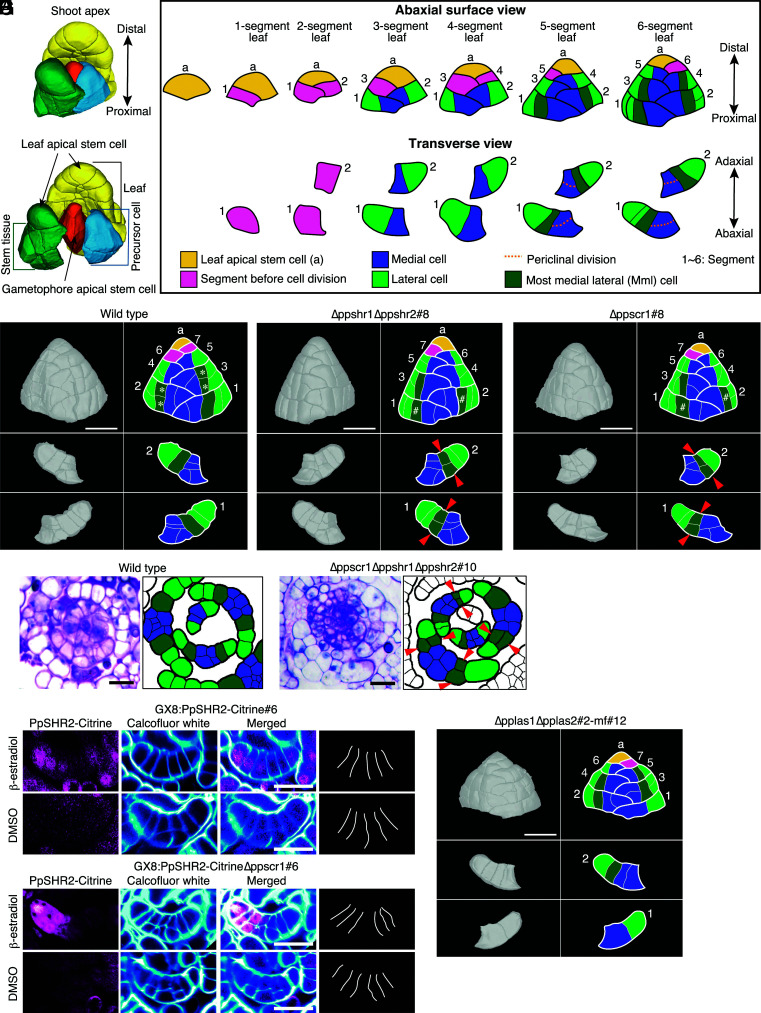
Regulation of periclinal cell divisions in leaf cells by PpSHR1, PpSHR2, PpSCR1, PpLAS1, and PpLAS2. (*A*) Schematic structure of a shoot apex in *P. patens* based on cellular segmentation using MorphoGraphX (39) with a series of optical sections. The central cell in orange is a gametophore apical stem cell. Daughter cells originating from the same precursor cell are shown in the same color. (*B*) Schematic abaxial surface view of developing wild-type leaves and transverse views of the first and the second segments. *Left* to *Right*: leaf apical stem cell, 1-segment leaf, 2-segment leaf, 3-segment leaf, 4-segment leaf, 5-segment leaf, and 6-segment leaf. Leaf apical stem cells (yellow), segments before cell division (pink), lateral cells (light green), medial cells (blue), and most medial lateral (mml) cells (dark green) are shown in different colors. Segments were numbered from oldest to the newest. Dotted orange lines in the 5- and 6-segment leaves indicate periclinal cell division planes. (*C–E*) Representative abaxial surface views of a 7-segment leaf and transverse distal surface views of the first and second segments in wild-type (*C*), Δppshr1Δppshr2#8 (*D*), and ∆ppscr1#8 (*E*) plants. Images from a series of optical sections of the 7-segment leaves were 3D-constructed using MorphoGraphX. Cells are colored as in *B*. Asterisks (*) (*C*) and hash marks (#) (*D* and *E*) indicate mml cells that divide anticlinally and periclinally, respectively. Red arrowheads (*D* and *E*) indicate daughter cells of periclinally divided mml cells. (Scale bar, 20 μm.) (*F*) Representative toluidine blue-stained transverse section of a shoot apex in wild-type and ∆ppscr1∆ppshr1∆ppshr2#10 plants. Representative leaves in the left photographs are traced in the right drawings. The colors of the cells in the right are the same as in *B*. Red arrowheads indicate daughter cells of the periclinally divided mml cells. (Scale bars, 20 µm.) (*G*) Representative optical transverse sections of young leaves in the PpSHR2–Citrine induction plants. Gametophores of GX8:PpSHR2–Citrine#6 and GX8:PpSHR2–Citrine∆ppscr1#6 plants were cultivated in the presence of 1 µM β-estradiol or DMSO for 8 d and stained with calcofluor white to visualize cell walls. Fluorescent images from a series of optical sections for each leaf were 3D-constructed to display the optical transverse sections (*H*) and paradermal sections (*SI Appendix*, Fig. S4*H*). Magenta and cyan indicate PpSHR2–Citrine and calcofluor white signals, respectively. Merged images are also shown. Schematic illustrations of representative leaves are shown at right, colored as in *B*. Red arrowheads indicate daughter cells of the periclinally divided mml cells. (Scale bar, 20 µm.) (*H*) Representative abaxial surface view of a 7-segment leaf and transverse distal surface views of the first and second segments in Δpplas1Δpplas2#2-mf#12 plant. Cells are colored as in *B*. (Scale bar, 20 µm.)

The gametophore apical stem cell produces a precursor cell that divides asymmetrically twice to form a leaf apical stem cell and other cells that become stem tissue (Fig. 2*A*) (38). The leaf apical cell repeatedly produces wedge-shaped cells called “segments” in the proximal direction (#1 to #6 pink cells in Fig. 2*B*). Each segment then divides longitudinally to produce a “medial cell” and a “lateral cell”. Subsequently, the medial and lateral cells divide periclinally and anticlinally, respectively. The anticlinal divisions include longitudinal or transverse divisions. We found an initial difference in division pattern between wild-type and Δppshr1Δppshr2 plants in the second cell division (Fig. 2 *C* and *D*). In wild type, the daughter cell of the lateral cell facing the medial cell, named the most-medial lateral (mml) cell, divided transversely and formed laminar tissue (n = 57) (white asterisks in Fig. 2*C*), whereas the corresponding cells in Δppshr1Δppshr2 usually divided periclinally to form midrib tissue (83 of 86 observed cells) (“#” in Fig. 2*D*), resulting in a wider midrib (Fig. 1 *B* and *C*). We also observed a periclinal cell division in some mml cells of the single deletion mutants (nine of 49 cells examined in Δppshr1 and nine of 59 cells in Δppshr2). These results indicate that *PpSHR1* and *PpSHR2* cause the mml cell to favor anticlinal rather than periclinal divisions.

**Fig. 3.**
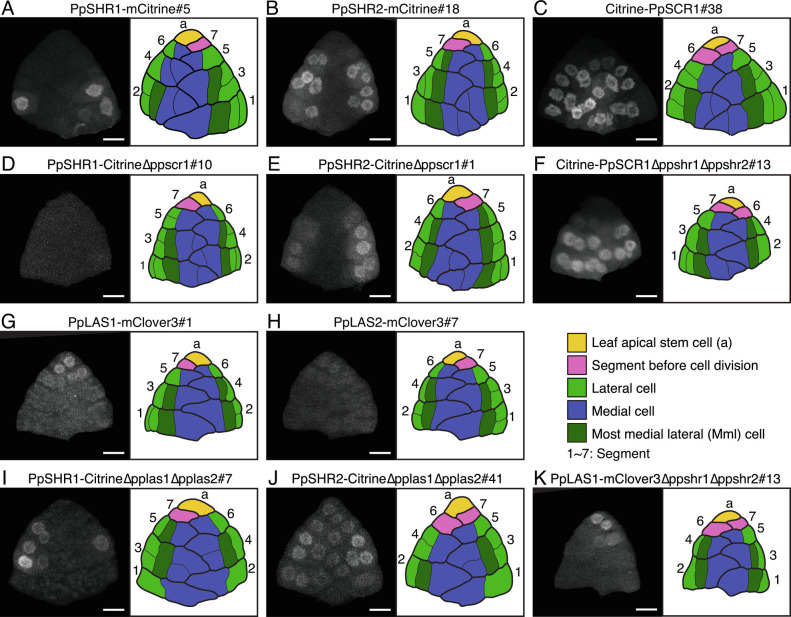
Accumulation of three GRAS transcription factors during leaf development. (*A–K*) Representative spatial accumulation patterns of three GRAS transcription factors fused to fluorescent proteins in 7-segment leaves. A 7-segment leaf in PpSHR1–mCitrine#5 (*A*), PpSHR2–mCitrine#18 (*B*), Citrine–PpSCR1#38 (*C*), PpSHR1–Citrine∆ppscr1#10 (*D*), PpSHR2–Citrine∆ppscr1#1 (*E*), Citrine–PpSCR1∆ppshr1∆ppshr2#13 (*F*), PpLAS1-mClover3#1 (*G*), PpLAS2-mClover3#7 (*H*), PpSHR1–Citrine∆pplas1∆pplas2#7 (*I*), PpSHR2–Citrine∆pplas1∆pplas2#41 (*J*), and PpLAS1-mClover3∆ppshr1∆ppshr2#13 (*K*) plants were stained with calcofluor white and observed with confocal microscopy. Fluorescent images from a series of optical sections for each leaf were constructed and are displayed after the sum projection. Schematic abaxial surface views of these leaves are also shown. Leaf apical stem cells (yellow), segments before cell division (pink), lateral cells (light green), medial cells (blue), and most medial lateral (mml) cells (dark green) are shown in different colors. (Scale bars, 10 µm.)

To further investigate *PpSHR* functions, we investigated the localization of PpSHR1 and PpSHR2 during leaf development. Transgenic lines were generated harboring the fluorescent reporter gene *mCitrine* (40) inserted just before the stop codon at the respective native *PpSHR* loci (*SI Appendix*, Fig. S3). We observed PpSHR1–mCitrine fluorescent signal in some lateral daughter cells (Fig. 3*A*). We also detected PpSHR2–mCitrine in the lateral but not the medial daughter cells (Fig. 3*B*). Importantly, we detected the PpSHR2 fusion protein in all mml cells and the PpSHR1 fusion protein in some mml cells (Fig. 3 *A* and *B*). This localization pattern and the phenotypes of the single deletion mutants supported the hypothesis that PpSHRs redundantly inhibit periclinal cell divisions in mml cells.

We also asked whether PpSHR can inhibit periclinal cell divisions in medial cells by ectopically inducing PpSHR2–Citrine accumulation in all leaf cells with the estrogen inducible system (41) (GX8:PpSHR2–Citrine; *SI Appendix*, Fig. S4). All new leaves formed after *PpSHR2–Citrine* induction appeared to lose midribs formed by periclinal cell divisions (Figs. 1*E* and 2*G* and *SI Appendix*, Fig. S4), indicating that *PpSHR2* inhibits periclinal cell divisions in both medial and mml cells. As Arabidopsis SHR moves between cells (18), we tested whether PpSHR2 is also a mobile protein by inspecting the accumulation of PpSHR2–mCitrine–GUS (a large protein fusion between PpSHR2, mCitrine, and β-glucuronidase [GUS]) and PpSHR2–mCitrine fusion proteins, whose expression was driven by the *PpSHR2* promoter (*SI Appendix*, Fig. S5). If it functions in a manner similar to Arabidopsis SHR and other mobile proteins, PpSHR2–Citrine would be expected to pass through plasmodesmata, whereas PpSHR2–mCitrine–GUS should be too large to do so. However, both fluorescent fusion proteins showed the same accumulation pattern, which was indistinguishable from that seen for nuclear localization signal (NLS)-enhanced green fluorescent protein–GUS fusion protein regulated by the *PpSHR2* promoter (PpSHR2pro:NLS–eGFP–GUS#28: *SI Appendix*, Fig. S5), indicating that the transcriptional and translational fusions show the same expression patterns. These results indicate that PpSHR2 does not move between cells.

### SCARECROW Coregulates the Inhibition of Periclinal Divisions with SHORTROOT.

In Arabidopsis roots, SHR regulates periclinal cell divisions of CEI cells by positively inducing *SCR* transcription (16, 17). Moody et al. identified three Physcomitrium orthologs of SCR and the closest homolog of SCR, SCARECROW-LIKE 23 (SCL23), in the genome (42) (*PpSCR1*, *PpSCR2*, and *PpSCR3*; *SI Appendix*, Fig. S6*A*). Reverse transcription quantitative PCR (RT-qPCR) indicated that *PpSCR1* is more abundant than the other *PpSCR*s in gametophores (*SI Appendix*, Fig. S6*B*), prompting us to focus on *PpSCR1*, although we cannot exclude possible involvement of *PpSCR2* and *PpSCR3* in this gene regulatory network.

To explore whether *PpSCR1* might be involved in the inhibition of periclinal cell divisions of mml cells, we knocked-in the *Citrine* gene in frame at the 5′ end of *PpSCR1* (*SI Appendix*, Fig. S7). We detected the Citrine–PpSCR1 fusion protein in most leaf cells, including mml cells (Fig. 3*C*). We also deleted *PpSCR1* in wild type and in the Δppshr1Δppshr2 mutant (*SI Appendix*, Fig. S8). In both of the resulting mutants (∆ppscr1 and ∆ppscr1∆ppshr1∆ppshr2), mml cells divided periclinally, as in the Δppshr1Δppshr2 double mutant (Fig. 2 *E* and *F*), indicating that PpSCR1 contributes to the regulation of cell division orientation in mml cells.

To investigate the regulatory relationship between PpSCR1 and PpSHRs, we determined the localization of PpSHR1–Citrine and PpSHR2–Citrine in the Δppscr1 mutant, as well as that of Citrine–PpSCR1 in the Δppshr1Δppshr2 double mutant (*SI Appendix*, Fig. S9). Neither PpSHR1–Citrine nor PpSHR2–Citrine accumulated in mml cells of the ∆ppscr1 mutant (Fig. 3 *D* and *E*), while the Citrine–PpSCR1 expression pattern was largely unchanged in mml cells in the ∆ppshr1∆ppshr2 double mutant (Fig. 3*F*). In a reciprocal approach, we induced the accumulation of PpSHR2–Citrine with β-estradiol, which did not affect relative *PpSCR* transcript levels, as shown by RT-qPCR (*SI Appendix*, Fig. S4). We also created transgenic plants with inducible expression of PpSCR1 (*SI Appendix*, Fig. S10). However, the induction of Citrine–PpSCR1 in the GX8:Citrine–PpSCR1 line arrested leaf development (*SI Appendix*, Fig. S10), thus preventing us from measuring relative *PpSHR* transcription levels. Together, these results indicate that PpSCR1 is necessary for the transcription of *PpSHR* genes in mml cells, which was in contrast to the reduction in SCR transcription observed upon mutating SHR in Arabidopsis (16).

We further investigated whether PpSCR1 is necessary to inhibit periclinal cell divisions in mml cells by inducing *PpSHR2* in the ∆ppscr1 mutant background and found that mml cells divided periclinally (Fig. 2*G*). This supported the hypothesis that PpSCR1 and PpSHR2 cooperate in the inhibition of periclinal cell divisions in mml cells.

As SCR and SHR form a heterodimer in Arabidopsis roots to induce *SCR* transcription (43), we assessed the interaction potential between PpSCR and PpSHRs. Indeed, we determined that PpSCR1 interacts with both PpSHR1 and PpSHR2 in yeast two-hybrid experiments (*SI Appendix*, Fig. S11), although coimmunoprecipitation assays using young gametophores were unsuccessful (*SI Appendix*, Fig. S12). It will be further investigated whether other PpSCRs interact with PpSHR2, since SHR also interacts with the closest homolog of SCR, SCL23, to specify endodermis cell fate in Arabidopsis roots (44).

### LATERAL SUPPRESSOR Promotes Periclinal Cell Divisions by Repressing SHORTROOT.

We detected Citrine–PpSCR1 in the medial cells undergoing periclinal cell divisions (Fig. 3*C*), in which neither PpSHR1–mCitrine nor PpSHR2–mCitrine accumulated (Fig. 3 *A* and *B*). This suggested that *PpSHR*s might be repressed in medial cells. When we analyzed a double deletion mutant for another pair of GRAS transcription factor genes, *Physcomitrium patens LATERAL SUPPRESSOR1* (*PpLAS1*) and *PpLAS2* (*SI Appendix*, Figs. S13 and S14), we discovered that the midrib of the double mutant was missing, as observed earlier in GX8:PpSHR2–Citrine following induction (Fig. 1 *E* and *F*). The recent study also showed the loss of midrib in the double mutant (45). In wild type, medial cells divided periclinally to form midrib tissue, while in the ∆pplas1∆pplas2 double mutant, the same cells divided transversely or longitudinally, but not periclinally (Fig. 2*H*). We then inspected the localization of PpLAS1–mClover3 and PpLAS2–mClover3 fusion proteins expressed from their endogenous loci, and found that they accumulate in the leaf apical stem cell and surrounding segments (Fig. 3 *G* and *H* and *SI Appendix*, Fig. S15). We also observed mClover3 florescence in both medial and lateral cells before the cell division that produces mml cells (5- and 6-segments in Fig. 3 *G* and *H*), but not in the cells of segments in which mml cells had been formed (3- and 4-segements in Fig. 3 *G* and *H*). These results suggested that PpLAS1 and PpLAS2 repress PpSHR accumulation. To directly test this possibility, we inserted *Citrine* in frame with each *PpSHR* endogenous locus in the Δpplas1Δpplas2 double mutant (*SI Appendix*, Fig. S16). Both PpSHR1–Citrine and PpSHR2–Citrine signals showed an expanded pattern, with PpSHR2–Citrine also detectable in medial cells (Fig. 3 *I* and *J*). Furthermore, ectopic induction of PpLAS1–Citirine in gametophores down-regulated the *PpSHR* expression using the GX8:PpLAS1–Citrine lines (*SI Appendix*, Fig. S17). The overexpression of PpLAS1–Citrine under the control of the native *PpLAS1* promoter activity in the ∆pplas1∆pplas2 double mutant (nPpLAS1pro:XVE>PpLAS1–Citrine∆pplas1∆pplas2) formed midrib tissue and narrow leaves, and had mml cells to divide periclinally, as in the Δppshr1Δppshr2 double mutant (*SI Appendix*, Fig. S17). These indicate that in the wild type, PpLAS1 negatively regulates PpSHR2 accumulation in medial cells (Fig. 3 *B* and *J*), resulting in periclinal division (Fig. 2*B*). Reciprocal regulation seemed unlikely, as the PpLAS1–mClover3 fluorescence intensity and pattern in the PpLAS1–mClover3 Δppshr1Δppshr2 line (Fig. 3*K* and *SI Appendix*, Fig. S16) were indistinguishable from those seen for PpLAS1–mClover3 in the wild-type background (Fig. 3*G*), suggesting that *PpSHR*s do not regulate PpLAS1 accumulation.

### GRAS Transcription Factors Change the Geometric Rule and Regulate Periclinal Cell Divisions.

**Fig. 4.**
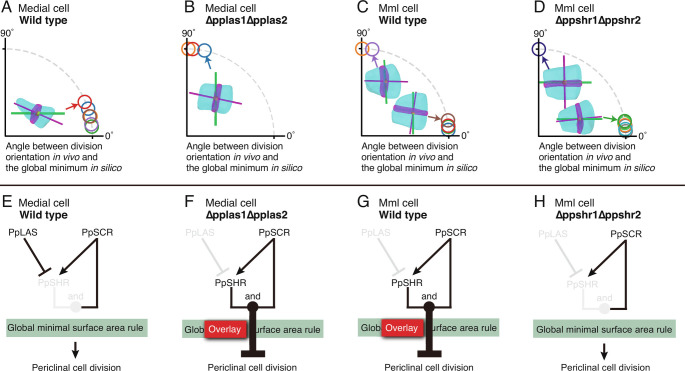
GRAS transcription factors overlay the geometric rule and prohibit periclinal cell divisions. (*A–D*) Polar plots indicating the angle (Eq. 9 in *Materials and Methods*) between the observed division orientation (horizontal axis; Eq. 1) and the simulated cell division orientation with global minimum surface area (Eq. 8). Representative cells are shown with the observed cell division orientation (green), the simulated cell division orientation (magenta) with the global minimum surface (magenta), and the centroid (yellow sphere) of mother cells (cyan). When the observed plane overlaps with the simulated plane, the circles are grouped close to the horizontal axis; when the observed plane is perpendicular to the simulated plane, the circles are grouped close to the vertical axis. Each circle indicates a different cell. (*B*) Two of the four circles almost overlap. (*E–H*) A genetic regulatory model for periclinal cell divisions in the medial and mml cells. Wild-type or GFP-α-Tubulin (*A*), ∆pplas1∆pplas2 (*B*), GFP-α-Tubulin (*C*), and GFP-α-Tubulin *∆*ppshr1∆ppshr2 (*D*) plants were observed.

The orientation of cell division during *Arabidopsis* early embryogenesis follows a geometric rule, whereby the smallest wall area passes through the cell centroid (46). However, embryos deviate from this rule during later developmental stages owing to the effects of regulatory mechanisms related to auxin (30). To determine if the geometric rule governed the orientation of cell division in medial and mml cells, we introduced a segmented three-dimensional cell shape (39), as well as the measured volume ratios of all cells, into the phase-field model (47). We then checked whether the model resulting from the observed orientations of cell division planes in segmented medial and mml cells coincided with the orientations of cell division planes expected if the geometric rule were satisfied. In wild-type medial cells in which PpSHRs did not accumulate, all observed division planes were periclinal and coincided with the simulated division planes with globally minimum areas (n = 6; Fig. 4*A* and *SI Appendix*, Fig. S19*A*). In Δpplas1Δpplas2 medial cells, in which PpSHRs accumulated, all observed division planes were transverse and did not coincide with those with global minimum areas (n = 4, Fig. 4*B* and *SI Appendix*, Fig. S19*B*). Based on these results, we hypothesized that PpSHRs override the geometric rule to prevent periclinal cell divisions (Fig. 4 *E* and *F*). Furthermore, in wild-type mml cells in which PpSHRs accumulate, none of the cells we examined divided periclinally. Instead, they divided transversely regardless of cellular geometry (four examined cells with global minimal division planes and two nonglobal minimal ones, Fig. 4*C* and *SI Appendix*, Fig. S19*C*), which was concordant with our hypothesis (Fig. 4*G*). Finally, mml cells in the Δppshr1Δppshr2 double mutant divided periclinally (n = 9), and their division planes coincided with those with the global minimum areas except in one examined cell with a nonglobal minimum area (Fig. 4*D* and *SI Appendix*, Fig. S19*D*). In this cell, the observed area was close to matching the expected area (4%, *SI Appendix*, Fig. S19*E*). Together, these results support the hypothesis that PpSHRs override the geometric rule to prevent periclinal cell divisions (Fig. 4 * E*-*H*).

### Physcomitrium SHRs Affect Transcript Levels of Genes Involved in the Cell Cycle.

To dissect the molecular mechanisms downstream of PpSHRs, we compared the transcriptomes in the apical part of gametophores including developing leaves between wild-type and *∆*ppshr1∆ppshr2 plants (Dataset S1). Unlike for the Arabidopsis SCR and SHR targets (19, 48), transcript levels of D-type cyclin genes were not significantly changed in *∆*ppshr1∆ppshr2. By contrast, transcript levels of two type-A cyclin (*CYCA1*) and both type-B cyclin (*CYCB2*) genes decreased significantly, by as much as ~1.5-fold, in *∆*ppshr1∆ppshr2, whereas none of the cyclin genes showed a significant increase (Dataset S2).

## Discussion

The orientation of divisions and the direction of growth in cells often affect development in multicellular organisms. Especially in plants, with their rigid cell wall, proper regulation of the orientation of cell divisions in both space and time is crucial for development. This study showed that the GRAS transcription factors PpSHR1 and PpSHR2 inhibit periclinal cell divisions of medial and mml cells during leaf vein formation of Physcomitrium and that two other GRAS factors, PpSCR1 and PpLASs, positively and negatively regulate PpSHR expression, respectively (Fig. 4). Repression of PpSHRs by PpLASs in medial cells is indispensable for leaf vein formation, in which water-conducting cells called hydroid cells are formed by programmed cell death (Fig. 1). In contrast to xylem vessel elements with secondarily thickened cell walls in vascular plants, hydroid cell walls are thinner than those of the surrounding cells. Xu et al. (2014) (49) showed that orthologous NAC domain transcription factors regulate programmed cell death in both Arabidopsis and Physcomitrium, but only in Arabidopsis were the genes involved in secondary cell wall thickening (49). This observation suggests that the regulatory network guiding the formation of water-conducting tissue evolved in the common ancestor of land plants, whereas wall thickening evolved in the angiosperm lineage after its divergence from the moss lineage (49). The formation of water-conducting tissue necessitates an increase in the number of cell layers, which are produced by periclinal cell divisions. We showed that such divisions are regulated by GRAS transcription factors in Physcomitrium as well as in Arabidopsis, suggesting the ancestral origin of the underlying regulatory mechanism.

While the same GRAS gene family is involved in periclinal divisions in both angiosperms and mosses, the regulatory networks differ between the two lineages. PpSHR2 in moss is not mobile and functions cell-autonomously, even though in Arabidopsis, SHR protein movement is key to forming different cell types in the root (18). In Arabidopsis roots, SHR movement is regulated by BIRD/INDETERMINATE DOMAIN zinc finger proteins and putative endosome-associated protein SHORTROOT INTERACTING EMBRYONIC LETHAL (SIEL) in addition to SCR and its paralog SCL23 (44, 50, 51). Future analyses of Physcomitrium orthologs to these movement regulators will give an insight into the evolution of SHR movement. Whereas SHR induces *SCR* transcription in Arabidopsis, *PpSCR1* transcript levels were unchanged upon PpSHR induction in Physcomitrium. Furthermore, *PpLAS*, whose ortholog is involved in lateral shoot formation independently of *SHR* and *SCR* in Arabidopsis, participates in the PpSCR1–PpSHR regulatory network of Physcomitrium. These distinct features may have produced the diversity of water-conducting systems between mosses and angiosperms. Besides roots, angiosperm GRAS genes are also involved in regulating cell divisions in other tissues including shoots (52–56). We observed that Δppshr and Δppscr1 mutants form narrower leaf blades than wild type (Fig. 1), indicating that Physcomitrium PpSHRs and PpSCR1 may also regulate cell division in other cell types, although it is unknown whether cell autonomous or not. In Arabidopsis leaves and hypocotyl, *SCL23* paralogous to *SCR* is involved in the SCR–SHR genetic regulatory network and functions in the differentiation of bundle sheath and endodermoidal cells, respectively (57, 58). Involvement of PpSCR in cellular differentiation was not recognized in this study. Further studies will be necessary to understand the full range of functions of PpSHRs and PpSCR1. It is noteworthy that GRAS gene family was horizontally transferred from soil bacteria to an aquatic ancestor of land plants and contributed to acquiring the indispensable function to survive on land.

In the absence of PpSHR expression, cells follow a cell division plane with the minimal surface area passing through the center of gravity, indicating that these cells divide according to the default geometric rule. In the wild type, PpSHR accumulation causes cells to deviate from the geometric rule and divide periclinally. The geometry-based rule can be bypassed by changing the cellular tensile stress caused by tissue deformation (22). Anisotropic stress on cells in the boundary region between the shoot meristem and the outgrowing primordium increases as cell shape changes and the cell grows, and the boundary cells divide according to the direction of tensile stress rather than according to the geometric rule (26). However, in the Physcomitrium leaf primordium at the stage analyzed in this study, medial and mml cells were the first cells to show different division orientations between wild type and the ∆ppshr1∆ppshr2 double mutant, whereas tissue tensile stresses did not appear to differ between the two genotypes according to their outer morphology (Fig. 2).

Cortical microtubules play a prominent role in guiding the orientation of cell division (59) by integrating local and global mechanical stress (60). The orientation of the mitotic spindle usually determines the division plane, which is predicted and stabilized by the preprophase band that forms in the cortical region through connection to the cortical microtubule network (61, 62). The origin of the default geometric rule used in land plants is enigmatic. Cell divisions of the Zygnematalean green algae, sister to land plants follow to the geometric rule with cortical microtubules and preprophase bands (63), while those of Coleochaete, sister to the land plants/Zygnematalean algae clade, follow to the default rule without cortical microtubules (27, 64). It is plausible that the geometric rule used in the common ancestor of land plants and Zygnematalean algae was overrode by GRAS and other factors to form complex tissue in land plants.

In Arabidopsis, the SHR/SCR protein complex promotes the expression of a D-type cyclin *CYCD6;1* in the CEI daughter cells, resulting in their periclinal cell division (19, 48). While transcript levels of D-type cyclin genes did not differ between *∆*ppshr1∆ppshr2 and wild type, two B-type cyclin genes, whose transcript levels increase during the G2/M phase (65), exhibited lower transcript levels in Δppshr1Δppshr2. Considering that Arabidopsis SCARECROW-LIKE 28 (66, 67), another GRAS family member, regulates cell cycle progression through G2/M and the orientation of cell division, Physcomitrium GRASs may function in the G2/M phase as well.

Horizontal gene transfer plays an adaptive role in the recipient lineages because of the acquisition of genes for de novo evolution (68). Horizontal gene transfer is popular in prokaryotes to acquire adaptive traits, such as antibiotic resistance and virulence (69). The significance of HGT in the terrestrialization of green plants also has been reported in some enzymes involved in the vascular cell differentiation (70) and DNA repair (71). Acquisition of GRAS genes from soil bacteria in the green algal ancestor of land plants has been discussed to have contributed the increase in resistance to biotic and abiotic stresses (6). This study implies that a horizontally transferred ancestral GRAS gene expanded in copy number in the genome of the land plant ancestor and formed a genetic regulatory network regulating periclinal cell divisions. This network was then further elaborated in each bryophyte and angiosperm lineage, while retaining orthologous genes. We propose that horizontally transferred genes contributed to the evolution of body plan in land plants through the regulation of cell division orientation.

## Materials and Methods

### Plant Materials, Culture Conditions, and Transformation.

The Cove-NIBB strain of *Physcomitrium patens* was used as the wild-type strain (72), and cultured on BCDAT or BCD agar medium at 25 °C under continuous white light or long-day (16-h light, 8-h dark) conditions (73). Transformation was performed by the polyethylene glycol-mediated method as described previously (73).

### Phylogenetic Analyses.

Methods for SHR, SCR, and LAS phylogenetic analyses are shown in legends of *SI Appendix*, Figs. S1, S6, and S13, respectively.

### Plasmid Construction.

Deletion and expression constructs for *PpSHR*s, *PpSCR1*, and *PpLAS*s were generated as shown in *SI Appendix*, Figs. S2–S5, S7–S10, S12, and S14–S18. Primers used for plasmid construction are listed in *SI Appendix*, Table S1.

### DNA Gel Blot Analysis.

DNA gel-blot analysis was performed as described previously (65).

### Histological Analysis.

Gametophores were fixed with 4.0% glutaraldehyde in 0.1 M phosphate buffer (pH 7.2) at 4 °C overnight and embedded in Technovit 7100 resin (Heraeus Kulzer). Five micrometer sections were cut and stained with 0.1% toluidine blue.

### Microscopy Analysis and Cell Segmentation.

Gametophores were cultivated for 3 wk on BCDAT medium under continuous light conditions. Mature leaves were removed for observation. To observe signals of fluorescent protein fused to GRASs, gametophores without mature leaves were fixed (4% [w/v] paraformaldehyde, 0.1% [w/v] glutaraldehyde, and 100 µg/mL calcofluor white M2R [Fluorescent Brightener 28; Sigma-Aldrich] in phosphate-buffered saline [PBS]) for 3 d at 4 °C, washed twice for 5 min each in PBS, and immersed in ClearSee solutions (10% [w/v] xylitol powder, 12.5% [w/v] sodium deoxycholate, 25% [w/v] urea in water) (74) at room temperature for 1 d. The ClearSee solution was replaced once with a fresh solution. An SP8 confocal microscope (LEICA) equipped with a white light laser, a 405-nm laser, and hybrid detectors, was used for the imaging with 40×, 1.10-NA and 63×, 1.20-NA water-immersion objective lenses. Fluorescent images were recorded at 0.18-µm or 0.25-µm intervals. To investigate whether the division planes follow to the geometric rule in mml cells, we selected cells with phragmoplasts in the GFP-α-Tubulin line as wild type and the GFP-α-Tubulin∆ppshr1∆ppshr2 line as ∆ppshr1∆ppshr2 mutant (*SI Appendix*, Fig. S18) to determine their cell shape, thereby eliminating any cell shape change that might have arisen from cell growth after cell division. In medial cells, it was not possible to observe more cells because of the difficulty to find cells with phragmoplasts in the medial cells. However, we inspected enough cells to establish that the orientation of cell division with global minimal area is periclinal rather than transverse, because of the rectangular cell shape that is longer along the adaxial–abaxial direction than in other directions. To observe microtubules in young leaves of the GFP-α-Tubulin#84 (GTU84) and GTU84∆ppshr1∆ppshr2#8 plants for the determination of cell division plane in mml cells, mature leaves were removed from gametophores of each plant, and the shoot apices were treated with fixative solution (8% [w/v] paraformaldehyde, 100 mM PIPES-NaOH, pH 6.8, 25 mM EGTA, pH 8.0, 1 mM MgCl_2_, 0.1% [w/v] glutaraldehyde, and 100 µg/mL Calcofluor white M2R) for 3 d at 4 °C.

Segmentation was performed using MorphoGraphX (39) (https://morphographx.org) based on fluorescence signal of calcofluor white. The segmented images were loaded to Fiji (https://imagej.net/software/fiji/) and used for masks to extract fluorescent protein signals of each cell or each leaf primordium.

### RT-qPCR Analysis.

For the quantification of *PpSCR1*, *PpSCR2*, and *PpSCR3* transcripts in gametophores, total RNA was purified from wild-type gametophores with the RNeasy Micro Kit (Qiagen). First strand cDNA was synthesized using ReverTra Ace qPCR RT Master Mix (TOYOBO). qPCR was performed using a QuantStudio 3 (ThermoFisher) instrument with the THUNDERBIRD SYBR qPCR Mix (TOYOBO). The primer sequences for RT-qPCR are described in *SI Appendix*, Table S3. The quantification of each sample was performed in technical triplicate and three biological replicates.

For the detection of the *PpSHR1*, *PpSHR2*, *PpSCR1*, *PpSCR2*, *PpSCR3* transcripts in plants expressing PpSHR2–Citrine or PpLAS1–Citrine, gametophores of the wild-type, GX8:PpSHR2–Citrine, GX8:NGG#4, and GX8:PpLAS1–Citrine plants were cultivated in liquid BCDAT medium with DMSO or 1 µM β-estradiol for 3 d. Purification of total RNA from each gametophore, first-strand cDNA synthesis, and RT-qPCR were performed as described above. The primer sequences for RT-qPCR are described in *SI Appendix*, Table S3. Results were analyzed using the comparative critical threshold method (59). The quantification of each sample was performed in technical triplicates. Three biological replicates were analyzed for the transcript accumulation.

### Transcriptome Analyses.

Apical parts of ten 3-wk-old gametophores from wild-type or ∆ppshr1∆ppshr2#8 plants were excised, and all visible leaves, except for youngest one, were removed using forceps under a stereomicroscope. The remaining parts, consisting of the gametophore apex with leaf primordia, the youngest developing leaf, and part of the stem, were immersed into 20 μL RLT buffer from the RNeasy Plant Mini Kit (Qiagen) with 0.2 μL β-mercaptoethanol and frozen in liquid nitrogen. Total RNA was extracted with an RNeasy Plant Mini Kit with DNase I treatment following the manufacturer’s protocol. cDNA was synthesized from 10 ng total RNA using SMART-Seq v4 (Takara) according to the manufacturer’s recommendations. cDNA sequencing libraries were prepared using the TruSeq Nano DNA Library Prep Kit (Illumina) and sequenced on a NextSeq500 (Illumina) platform following the manufacturer’s protocol. Single-end reads were preprocessed with cutadapt 2.8 (75) to remove adapter sequences and filter low-quality bases. The filtered reads were mapped to the Physcomitrium v3.3 transcriptome (Phytozome v12.1) using salmon 1.1.0 (76). Transcript level pseudocounts were imported into R Statistical Software (v4.0.3) and converted to gene level counts using the tximport package (77). Differentially expressed genes between wild type and ∆ppshr1∆ppshr2 were identified using DESeq2 with the Wald test (78) from three biological replicates.

### Yeast Two-Hybrid Assay.

The full-length cDNAs without the stop codon for *PpSHR1*, *PpSHR2*, and *PpSCR1* were RT-PCR amplified from wild-type gametophore, and cloned into the Gateway cloning vector pDONR22. After confirmation by sequencing, the cDNAs were subcloned into pDEST-BD and pDEST-AD vectors. GAL4–DNA binding domain- and GAL4-activation domain-fusion constructs were then transformed into competent yeast cells mav103 and mav203, respectively, which were mated by mixing the two strains to obtain diploid cells that express both fusion proteins. Primers used for plasmid construction are listed in *SI Appendix*, Table S4.

### Immunoprecipitation.

For protein–protein interactions in gametophores, total protein extracts from gametophores of PpSHR1-3×Myc#16, PpSHR2-3×Myc#46, Citrine–PpSCR1 PpSHR1-3×Myc#29, and Citrine–PpSCR1 PpSHR2-3×Myc#46 plants were prepared with immunoprecipitation buffer (25 mM Tris-HCl [pH 7.5], 75 mM NaCl, 15 mM MgCl_2_, 15 mM EGTA, 0.1 mM NP-40, 10 mM NaF, 25 mM β-glycerophosphate, 2 mM sodium *o*-vanadate, and 1× complete protease inhibitor cocktail [Roche]) and were immunoprecipitated with monoclonal anti-Myc (4A6; Sigma-Aldrich) or anti-GFP (JL-8; Clontech) antibodies. The crude extracts and immunoprecipitates were examined using anti-Myc and anti-GFP antibodies.

### Division Plane Quantification.

We calculated the orientation of the observed division plane by the normal vector $\hat{n}$:

[1] $$\hat{n}=\frac{\sum_{r\;r\;'\;}\delta_{1m\left(r\right)}\delta_{2m\left(r\;'\right)}\frac{r'-r}{\left|r',-,r\right|}}{\sum_{r\;r\;'\;}\delta_{1m\left(r\right)}\delta_{2m\left(r\;'\right)}},$$

where ***r*** and ***r’*** denote the position of the segmented daughter cells 1 and 2, respectively. *m*(***r***) denotes the daughter cell occupying ***r*** and takes either 0, 1, or 2, where *m*(***r***) = 0 denotes the extracellular space. The summations of ***r’*** and ***r*** were performed over the nearest six sites. $\hat{n}$ and $-\hat{n}$ are indistinguishable.

### Numerical Calculations of the Area-Minimized Division Plane.

We numerically obtained the division plane surfaces with the global or local minimum using the phase-field method (47). We used the observed three-dimensional segmented region of the mother cell (i.e., wild-type or Δpplas1Δpplas2 medial cell, wild-type or Δppshr1Δppshr2 mml cell). We then numerically calculated the region of the two daughter cells (*ρ_i_*(***r***, *t*), *i* = 1, 2) where the interfacial surface area of the cells was minimized, by a dynamical equation:

[2] $$\begin{array}{c} \left(\partial ,\rho_{i}\right)/\partial t=-\delta F[\rho_{1},\rho_{2}]/(\delta \rho_{i})+g_{i}(\rho_{i})\\ \;=\varepsilon^{2}\nabla^{2}\rho_{i}+\rho_{i}(1-\rho_{i})\\ \;(\rho_{i}-\frac{1}{2}+\alpha \left(V_{i,0},-,V,\left(\rho_{i}\right)\right))-\beta \rho_{i}\rho_{j}^{2}, \end{array}$$

where *ρ_i _*= 1 and *ρ_i _*= 0 represent the interior and exterior of daughter cell *i*, respectively, and *ρ_i_* varies smoothly at the cell membrane (interface). *j* is 2 for *i* = 1 and *j* is 1 for *i* = 2. *F*[*ρ*_1_, *ρ*_2_] denotes the total free energy defined as:

[3] $$F\left[\rho_{1},\;\rho_{2}\right]=\int_{\Omega }\sum_{i}f_{i}\left(\rho_{1},\;\rho_{2}\right)dr,$$

where Ω is the calculation region effectively corresponding to the mother cell region and *f_i_* denotes the free energy density defined as:

[4] $$f_{i}=\frac{\varepsilon^{2}}{2}{\left|\nabla \rho_{i}\right|}^{2}+\frac{1}{4}\rho_{i}^{2}{\left(1-\rho_{i}\right)}^{2}+\frac{\beta }{2}\rho_{i}^{2}\rho_{j}^{2}.$$

We introduced $g_{i}\left(\rho_{i}\right)=\alpha \rho_{i}\left(1-\rho_{i}\right)\left(V_{i,0}-V\left(\rho_{i}\right)\right)$ in Eq. 2 as the force imposing the cell volume constraint *V_i,_*_0_ = *V*(*ρ_i_*(***r***, *t*)), where *V*(*ρ_i_*(***r***, *t*)) and *V_i,_*_0_ denote the daughter cell volume and the target volume, respectively. We calculated Eq. 2 by the explicit Euler-scheme with *dx* = 0.05, ε = 0.045, *dt* = (*dx* / ε)^2^ / 30, α = 2 and β = 1, until it reached the steady state. To avoid delays and dead ends in the calculations due to the interface thinning of phase-fields *ρ_i_* (79, 80), we set target volume *V_i,_*_0_ to 90% of the observed volume in vivo. We set the initial condition of interface as a plane, which passes through the centroid and is perpendicular to one of the three axes of the eigenvectors of the inertial moment of the mother cell:

[5] $$I=\sum_{r}\left(\delta_{1m\left(r\right)}+\delta_{2m\left(r\right)}\right)r⊗r.$$

The volume of daughter cells was initially set as the target volume *V*(*ρ_i_*(***r***, 0)) = *V_i,_*_0_. We calculated the surface area of the division plane is as:

[6] $$S=\frac{1}{2}\left(S_{1}\;+\;S_{2}\;-\;S_{0}\right),$$

where *S_0_* is the surface area of the mother cell and *S*_1_ and *S*_2_ are the surface areas of the daughter cells. Each surface area was evaluated according to a previously reported formula (81):

[7] $$\begin{array}{c} S_{i}=\int_{\Omega }\left[\frac{\varepsilon {\left|\nabla \rho_{i}\right|}^{2}}{2}+\frac{\left({\rho_{i}}^{2}-1\right){\rho_{i}}^{2}}{4\varepsilon }\right]dr\;\;\;\;\;\;\;\;\left(i=0,\;1,\;2\right). \end{array}$$

This quantity provides both global and local minimal smooth surfaces (82). We calculated the minimal division plane orientation in simulations as the mean normal vector of the surface ${\hat{n}}_{\mathrm{num}}$:

[8] $$\begin{array}{c} {\hat{n}}_{num}=-\frac{\int_{\text{Ω}}(\nabla \rho_{1})\rho_{1}(1-\rho_{1})\rho_{2}(1-\rho_{2})dr}{\left|\int_{\text{Ω}}(\nabla \rho_{1})\rho_{1}(1-\rho_{1})\rho_{2}(1-\rho_{2})dr\right|}. \end{array}$$

The angular difference between the observed division plane orientation (Eq. 1) and simulated one (Eq. 8) was measured as (Fig. 4 *A*, *B*, *C*, and *D*):

[9] $$\Delta \theta =\arccos(\hat{n}\cdot {\hat{n}}_{\mathrm{num}}).$$

## Supplementary Material

Appendix 01 (PDF)

Dataset S01 (XLSX)

Dataset S02 (XLSX)

## Data Availability

All study data are included in the article and/or *SI Appendix*. RNA-seq data were deposited to DDBJ Sequence Read Archive (DRA) with the accession number DRA013762. The moss lines and plasmids used in this study will be shared upon request.
