# Supplementary material for: GRAS transcription factors regulate cell division planes in moss overriding the default rule
Source: Proc Natl Acad Sci U S A. 2023 Jan 20;120(4):e2210632120. doi: 10.1073/pnas.2210632120 (PMC9942845; doi:10.1073/pnas.2210632120)
Supplement: Supplementary file 1 — Appendix 01 (PDF) [file pnas.2210632120.sapp.pdf]

PNAS

Supplementary Information for

Three GRAS transcription factors regulate cell division planes with overlaying the default rule.

Masaki Ishikawa, Ayaka Fujiwara, Ken Kosetsu, Yuta Horiuchi, Naoya Kamamoto, Naoyuki Umakawa, Yosuke Tamada, Liechi Zhang, Katsuyoshi Matsushita, Gergo Palfalvi, Tomoaki Nishiyama, Sota Kitasaki, Yuri Masuda, Yoshiki Shiroza, Munenori Kitagawa, Toru Nakamura, Hongchang Cui, Yuji Hiwatashi, Yukiko Kabeya, Shuji Shigenobu, Tsuyoshi Aoyama, Kagayaki Kato, Saiko Yoshida, Takashi Murata, Koichi Fujimoto, Philip N. Benfey, Mitsuyasu Hasebe, Rumiko Kofuji

To whom correspondence may be addressed. Email: ishikam@nibb.ac.jp., fujimoto@bio.sci.osaka-u.ac.jp, kofuji@staff.kanazawa-u.ac.jp, mhasebe@nibb.ac.jp, philip.benfey@duke.edu

This PDF file includes:

Figures S1 to S19
Tables S1 to S4
SI References

Other supplementary materials for this manuscript include the following:

Datasets S1 and S2

Supplementary Figures

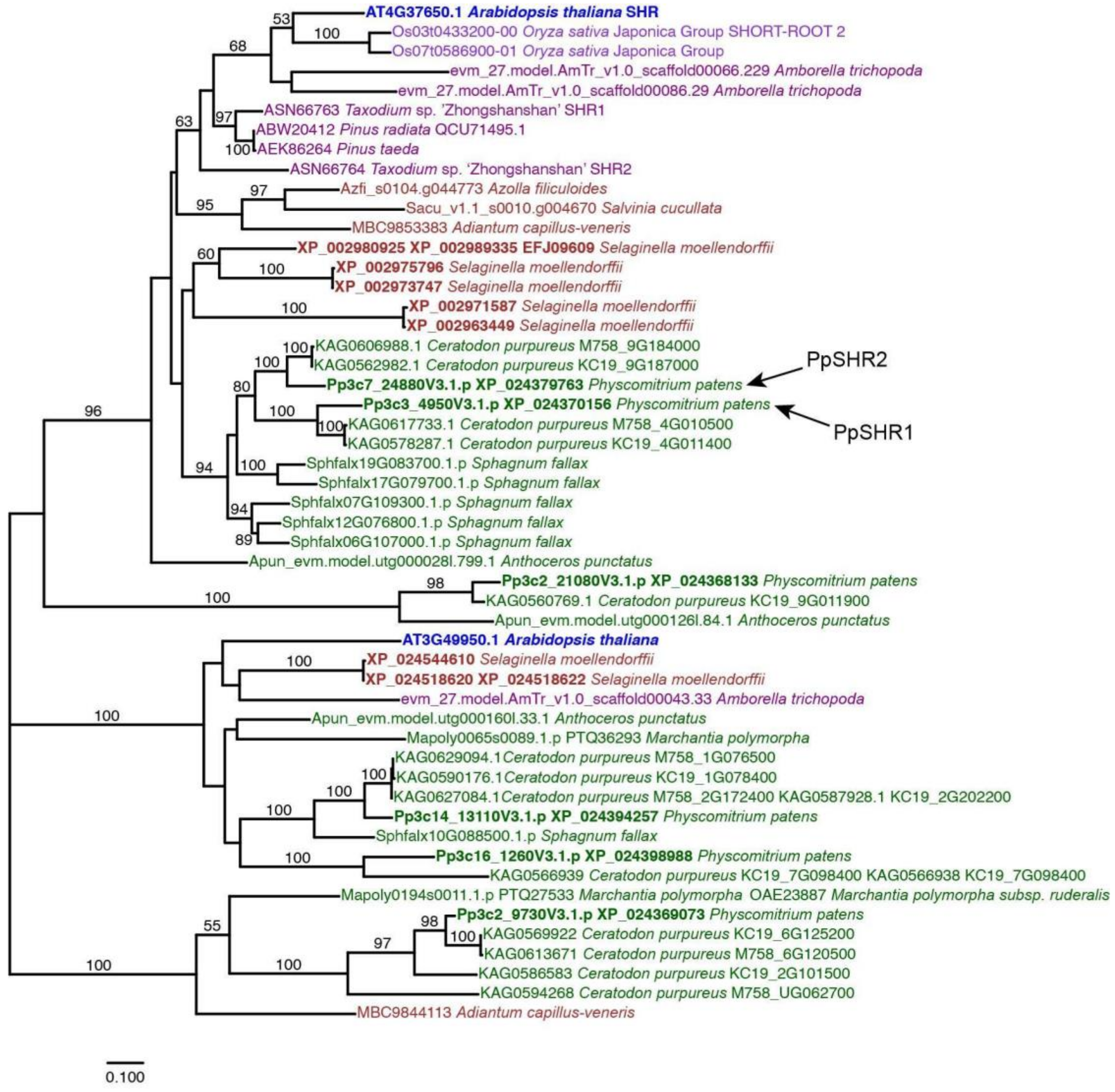

Fig. S1. Phylogeny of SHR in land plants.
A phylogenetic tree of SHR homologs in land plants. The two *Physcomitrium patens* proteins PpSHR1 and PpSHR2 are indicated with arrows. The maximum likelihood tree under the LG model (1) with empirical amino acid frequency was searched with RAxML-HPC. The bootstrap probabilities (%) based on 1,000 resamplings are shown on the branches where bootstrap probabilities were over 50%. The horizontal branch lengths are proportional to the estimated number of substitutions per site and are drawn to scale. INSD (International Nucleotide Sequence Databases) accession numbers or genome-specific identifiers are shown with the species name in which the sequence was present, sometimes followed by the protein name. Operational taxonomic units (OTUs) are color coded according to the classification: blue, eudicots; blue violet, monocots; dark magenta, other seed plants; brown, other vascular plants (monilophytes and lycophytes); dark green, bryophytes.

SHR homologs were searched from representative genome collections and nonredundant (nr) databases with restriction to the Viridiplantae but excluding angiosperms. The representative genome collection consisted of "Araport11" (*Arabidopsis thaliana* from TAIR), "Atrichopoda1" (*Amborella trichopoda* v 1.0 from Phytozome), "Mpo31" (*Marchantia polymorpha* v 3.1 from Phytozome), "Pita_2_01" (*Pinus taeda* v 2.01, Pita.2_01.peptides.fa.gz on Dec 28 2017 containing 36,732 sequences), "Ppa33" (*Physcomitrella patens* v 3.3

from Phytozome), "rice-IRGSP-1.0" (IRGSP-1.0_protein_2017-08-04.fasta), "Azolla" (*Azolla fillicoides* v1.1 from fernbase [https://www.fernbase.org]), "Salvinia" (*Salvinia* v 1.2 from fernbase), and "Selmo_ncbi" (GCF_000143415.4_v1.0_protein.faa). *Sphagnum fallax* v1.1 (DOE-JGI, http://phytozome.jgi.doe.gov/) was additionally searched at the Phytozome 13 site. The *Anthoceros punctatus* dataset was downloaded from https://www.hornworts.uzh.ch/en/download.html. BLASTP searches (2) were performed with a minimum word size of 2 and allowing up to 1,000 hits; the top 100 target amino acid sequences were then recovered. Thus, the SHR dataset of 100 representative plants + 100 non-angiosperm plants + 47 *Anthoceros punctatus* + seven *Sphagnum* entries was obtained.

The entire dataset was aligned with einsi in MAFFT version 7.475 (3). The alignment was imported into Mesquite version 3.61 (4). Redundant entries were not added as *Selaginella* sequences were contained in both the representative genome set and the non-angiosperm nr dataset. All characters in the alignment were first set as “excluded”. The alignments were then reviewed on Mesquite, resulting in the identification of well-aligned regions later set for “included”. Sequences harboring large deletions in the conserved region were removed, yielding a SHR data matrix with 203 entries, for each of which a maximum likelihood tree was constructed as follows: after examining the result for each of the 203 entries, the group derived from a protein encoded by a single gene in the last common ancestor of land plants was estimated, a smaller dataset comprising that group and two outgroup clades was chosen (74 entries for SHR), and the alignment was reinvestigated for inclusion/exclusion. Each data matrix was saved as a nexus file and subjected to a custom maximum likelihood analysis pipeline involving RAxML-HPC (5). The “included” sites were extracted from the matrix, and sequences identical in the included regions were treated as a single OTU during the analysis and reverted to the original names at the final stage. The extracted matrix was converted to PHYLIP format. A molecular evolutionary model was chosen using ProteinModelSelection.pl, and in this case, LGF (LG+F) was chosen for others. LG stands for the amino acid substitution matrix according to Le and Gascuel (2008) (1) and F stands for using empirical amino acid frequency in the dataset. SEQBOOT (6) was used to prepare 1,000 resampling sets. The original and bootstrap replicates were individually processed with the “-f a -# 100” option of RAxML. The model was specified with “-m PROTGAMMALGF” according to the chosen model. Random number seeds -x and -p option were taken from the operating system with /dev/urandom. After all jobs had finished, the resulting trees were recovered and the bootstrap frequency was calculated using CONSENSE (6). To force the topology to be the same as that of the tree obtained from the original dataset, the original tree was amplified 1,000 times and combined with the bootstrap results; after consensus calculation, 1,000 was subtracted from the result.

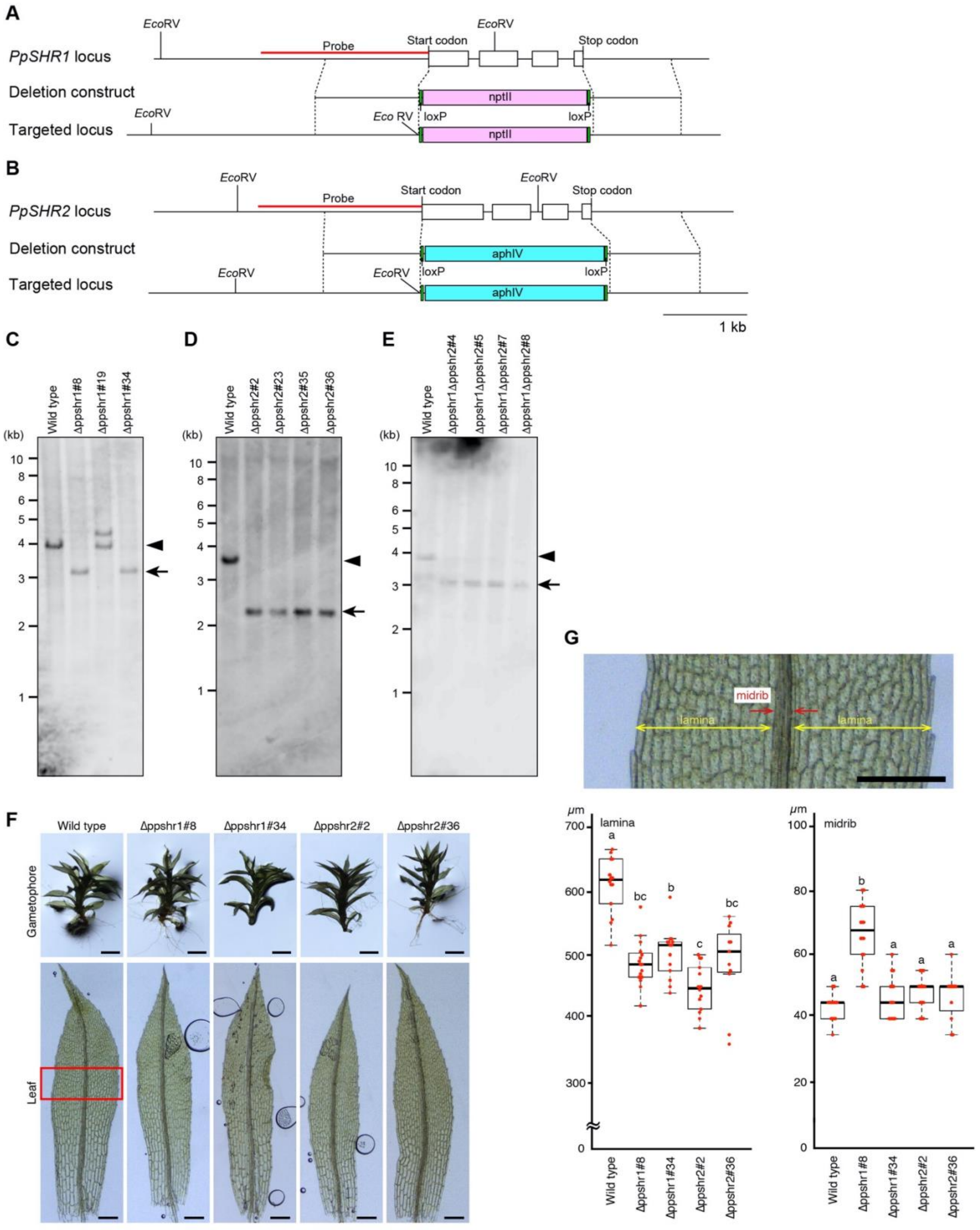
A
EcoRV
EcoRV
Probe
Start codon
Stop codon
PpSHR1 locus
Deletion construct
nptII
loxP
loxP
Targeted locus
EcoRV
Eco RV
nptII
B
EcoRV
EcoRV
Probe
Start codon
Stop codon
PpSHR2 locus
Deletion construct
aphIV
loxP
loxP
Targeted locus
EcoRV
EcoRV
aphIV
1 kb
C
Wild type
Δppshr1#8
Δppshr1#19
Δppshr1#34
(kb)
D
Wild type
Δppshr2#2
Δppshr2#23
Δppshr2#35
Δppshr2#36
E
Wild type
Δppshr1Δppshr2#4
Δppshr1Δppshr2#5
Δppshr1Δppshr2#7
Δppshr1Δppshr2#8
F
Wild type
Δppshr1#8
Δppshr1#34
Δppshr2#2
Δppshr2#36
Gametophore
Leaf
G
midrib
lamina
lamina
μm
lamina
μm
midrib

Fig. S2. Construction of the Δppshr1, Δppshr2, and Δppshr1Δppshr2 deletion plants.
(A and B) Schematic diagrams of the constructs targeting the *PpSHR1* (A) and *PpSHR2* (B) loci. White boxes represent exons. Magenta, cyan, and green boxes denote the neomycin phosphotransferase II expression cassette (nptII) (7), the aminoglycoside phosphotransferase IV expression cassette (aphIV) (8), and loxP sequences (9), respectively. Probes used in (C-E) are shown as red horizontal lines. To generate the *PpSHR1* and *PpSHR2* gene deletion constructs, genomic fragments containing the 5' and 3' flanking regions of each *PpSHR* gene were amplified and inserted into the 5' and 3' ends of the nptII expression cassette (A) in plasmid pTN182 (AB267706) or the aphIV expression cassette (B) in plasmid pTN186 (AB542059), respectively. The generated constructs were digested with suitable restriction enzymes for gene targeting and introduced into the wild type to generate Δppshr1 and Δppshr2 single deletion mutant plants. To generate Δppshr1Δppshr2 double deletion mutant plants, the *PpSHR1*-deletion construct was introduced into Δppshr2 plant #36.
(C-E) DNA gel blot analyses of targeted plants with the probes indicated in (A and B). Genomic DNA of wild-type, Δppshr1 (#8, #19, and #34), Δppshr2 (#2, #23, #35, and #36), and Δppshr1Δppshr2 (#4, #5, #7, and #8) plants was digested with *Eco*RV. Arrowheads and arrows indicate DNA fragments specific to wild-type and deletion mutant plants, respectively.
(F) Representative gametophores and leaves of Δppshr1 and Δppshr2 single deletion mutants and wild type. The area delineated by a red box is shown at higher magnification in (G). (Scale bars, 1 mm in upper panels, 200 µm in lower panels.)
(G) Quantification of the lengths of lamina and midrib in wild-type, Δppshr1, and Δppshr2 leaves. A magnified image of the wild-type leaf in (F) is shown above the plot. Yellow and red arrows indicate lamina and midrib, respectively. (Scale bar, 200 µm.) Lowercase letters indicate significant differences (one-way ANOVA and Tukey's test, $p < 0.05$).

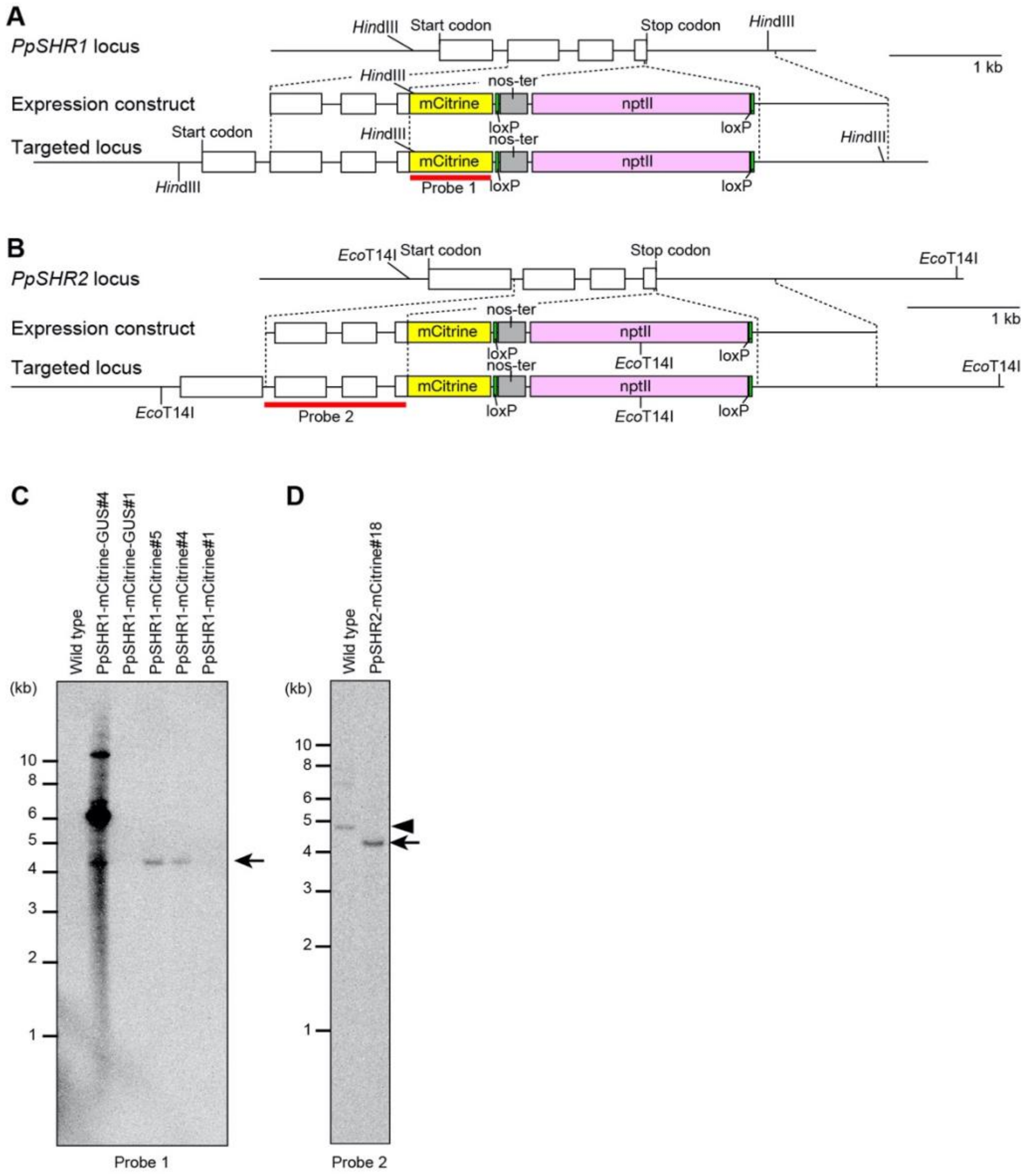

Fig. S3. Construction of PpSHR1-mCitrine and PpSHR2-mCitrine plants.
(A and B) Schematic diagrams of the targeting strategy for the *PpSHR1* (A) and *PpSHR2* (B and C) loci. White boxes represent exons. Yellow and magenta boxes denote *mCitrine* (10) or *Citrine* (11) and the neomycin phosphotransferase II expression cassette (nptII) (7), respectively. Green and gray boxes denote the loxP sequence (9) and the terminator from nopaline synthase (nos-ter) (7). Probes used in (C and D) are shown as red horizontal lines. To insert *mCitrine* in frame with the *PpSHR1* (A) or *PpSHR2* (B) coding sequences, a genomic DNA fragment for each locus extending from the middle to the last codon (5' fragment) and containing the 3' flanking region of each gene (3' fragment) was PCR amplified from wild-type genomic DNA and inserted into the *Hin*dIII and *Spe*I sites of the pmCit-nptII plasmid (ON092626), using the In-Fusion HD Cloning Kit (Takara). To insert *Citrine* in frame with the *PpSHR2* coding sequence, a genomic DNA fragment from the start codon to the last codon of the *PpSHR2* coding sequence, a DNA fragment encoding Citrine, a genomic fragment of the 3' flanking region of *PpSHR2*, and pGEM-3Zf(+) (Promega) were individually PCR amplified and precisely assembled using the GENEART Seamless Cloning and Assembly Kit (Thermo Fisher Scientific). The generated constructs were digested with suitable restriction enzymes for gene targeting and introduced into wild-type plants.

(C and D) DNA gel blot analyses of candidate targeted lines. (C) Genomic DNA of wild-type and PpSHR1-mCitrine (#1, #4, and #5) plants was digested with *Hin*dIII*.* PpSHR1-mCitrine-GUS (#1 and #4) plants were not used in this study. The arrow indicates DNA fragments specific to PpSHR1-mCitrine plants. (D) Genomic DNA of wild-type and PpSHR2-mCitrine#18 plants was digested with *Eco*T14I. The arrowhead and arrow indicate DNA fragments specific to wild-type and PpSHR2-mCitrine#18 plants, respectively.

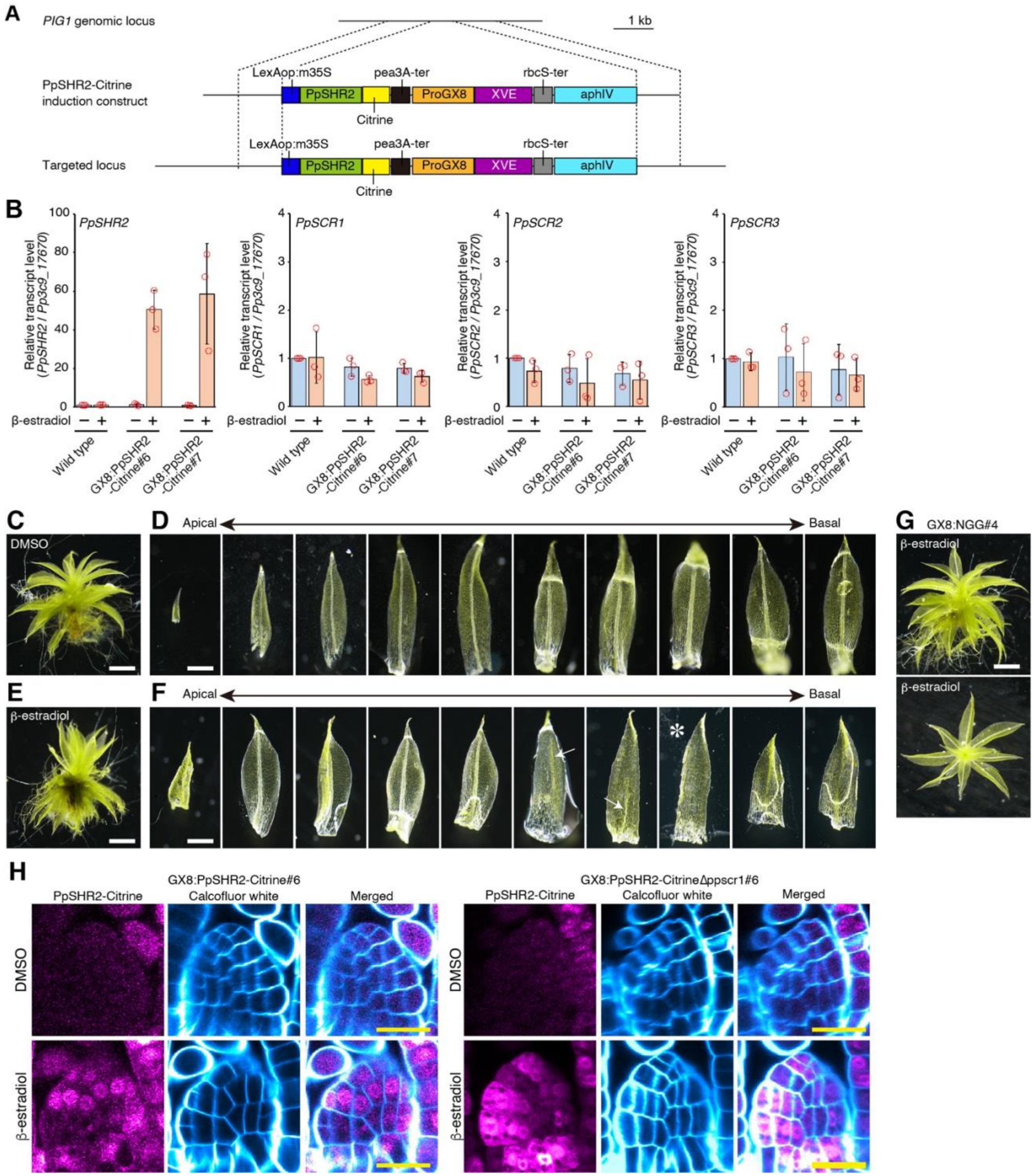

Fig. S4. Construction of GX8:PpSHR2-Citrine plants and PpSHR2-Citrine induction in gametophores.
(A) Schematic diagram showing the insertion of the *PpSHR2* induction construct into the *PIG1* putative neutral genomic locus (12). A connected DNA fragment of the *LexA* operator and minimal *35S* promoter (LexAop:m35S: blue) (13), *PpSHR2* (green), *Citrine* (yellow) (11), the pea rbcS3A terminator (pea3A-ter: black) (14), the putative promoter of *ProGX8* (orange) (12), a DNA fragment encoding an XVE fusion protein derived from pER8 (purple) (13), the *rbcS* terminator (gray: rbcS-ter) (14), and the aminoglycoside phosphotransferase IV expression

cassette (aphIV: cyan) (8) are shown in different colors. To produce the β-estradiol-inducible *PpSHR2-Citrine* induction construct, the *PpSHR2* coding sequence, a DNA fragment encoding Citrine, and the pENTR/D-TOPO vector were PCR amplified and precisely assembled by the GENEART Seamless Cloning and Assembly Kit (Thermo Fisher Scientific) to generate pENTR:PpSHR2cDNA-Citrine. The pENTR:PpSHR2cDNA-Citrine plasmid was subjected to LR reaction using the destination vector pPGX8 (AB537482) (12) to generate the GX8:PpSHR2-Citrine plasmid. The generated construct was digested with *Pme*I for gene targeting and introduced into wild-type plants.

(B) Induction of *PpSHR2-Citrine* in gametophores of GX8:PpSHR2-Citrine plants. Gametophores of wild-type, GX8:PpSHR2-Citrine#6, and GX8:PpSHR2-Citrine#7 plants were cultivated in liquid BCDAT medium with or without 1 µM β-estradiol for three days. Total RNA was purified from gametophores for RT-qPCR analysis. Relative *PpSHR2*, *PpSCR1*, *PpSCR2*, and *PpSCR3* transcript levels were obtained by normalization to that of *Pp3c9_17670* transcript encoding a thiosulfate sulfurtransferase (15); the value for each transcript in wild-type plants without β-estradiol was set to 1.0. The data points shown in red circles are the averages of three technical replicates in each plant, and the bars indicate the means of the relative transcript levels from three biological replicates. Error bars indicate standard deviations (SD).

(C-G) Representative gametophores of the GX8:PpSHR2-Citrine#6 and GX8:NGG#4 plant transiently treated with DMSO or 1 µM β-estradiol. Gametophores of the GX8:PpSHR2-Citrine#6 and GX8:NGG#4 plants grown on the solid BCDAT medium for 21 days after propagation were cultivated in the liquid BCDAT medium with DMSO (C) or 1 µM β-estradiol (E and G) for 7 days. The chemical-treated gametophores were washed with sterilized milliQ water and further cultivated on solid BCDAT medium for 10 days. Leaves were detached from the upper half of each gametophore and arranged in order from apical to basal (D and F). An asterisk and arrows in F indicate a leaf without midrib and partially formed midribs, respectively. Side and top views of a representative gametophore of the GX8:NGG#4 plant transiently treated with β-estradiol in the same conditions are also shown (G). (Scale bars, 1 mm in C, E, and G, 400 µm in D and F.)

(**H**) Representative optical paradermal sections of young leaves in the PpSHR2-Citrine induction plants. The same 3D-constructed images in Fig. 2G were used to display the optical longitudinal sections. Magenta and cyan indicate PpSHR2-Citrine and calcofluor white signals, respectively. Merged images are also shown. (Scale bar, 20 µm.)

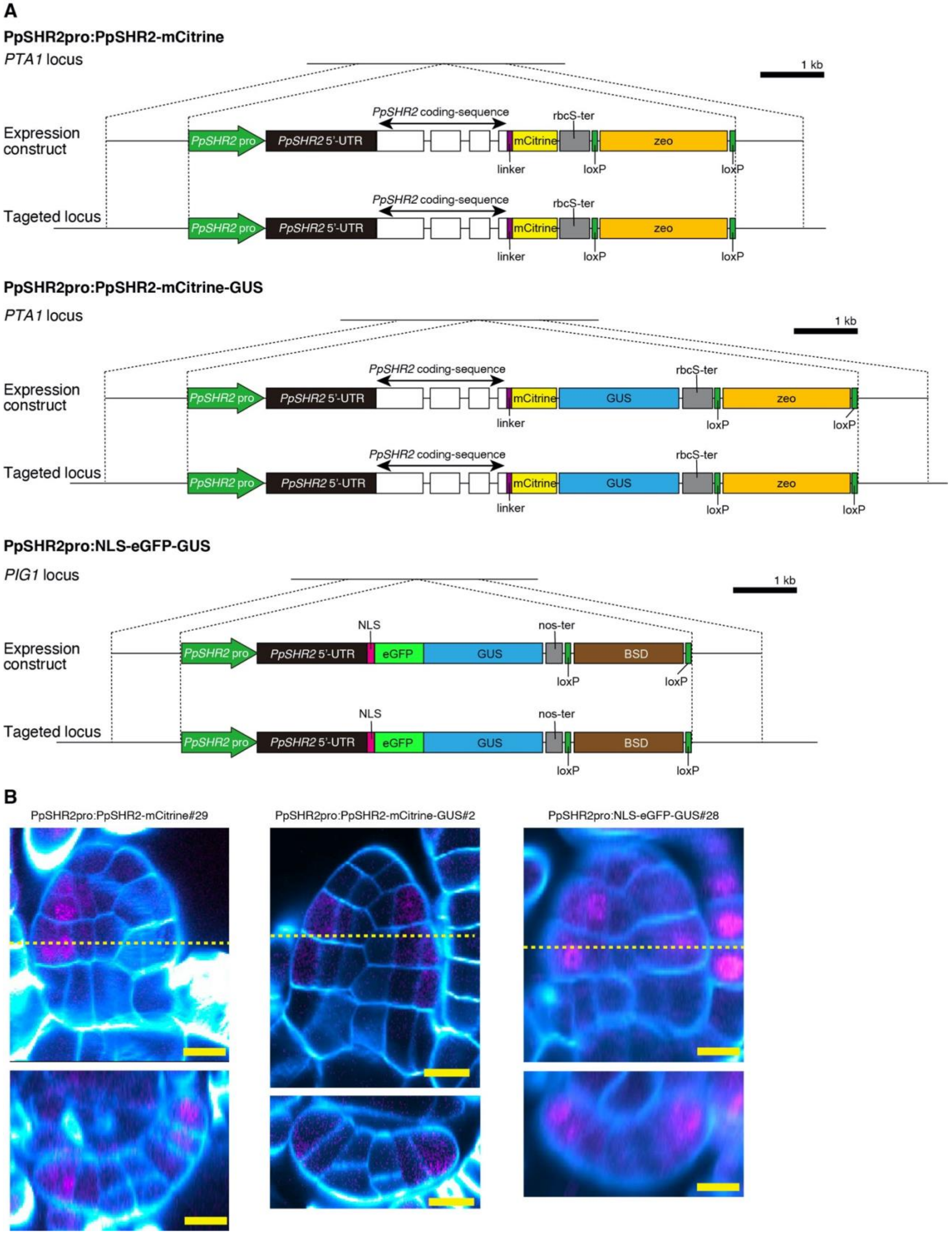

A
PpSHR2pro:PpSHR2-mCitrine
PTA1 locus
1 kb
Expression construct
Tageted locus
PpSHR2 coding-sequence
rbcS-ter
PpSHR2 pro
PpSHR2 5'-UTR
mCitrine
zeo
linker
loxP
PpSHR2pro:PpSHR2-mCitrine-GUS
PTA1 locus
GUS
PpSHR2pro:NLS-eGFP-GUS
PIG1 locus
NLS
nos-ter
eGFP
BSD
B
PpSHR2pro:PpSHR2-mCitrine#29
PpSHR2pro:PpSHR2-mCitrine-GUS#2
PpSHR2pro:NLS-eGFP-GUS#28

Fig. S5. Localization of PpSHR2-mCitrine, PpSHR2-mCitrine-GUS, and NLS-eGFP-GUS encoded by expression cassettes driven by the *PpSHR2* promoter.

(A) Schematic diagrams showing the insertion strategy of the PpSHR2pro:PpSHR2-mCitrine and PpSHR2pro:PpSHR2-mCitrine-GUS constructs into the *PTA1* genomic locus (12) and the PpSHR2pro:NLS-eGFP-GUS construct into the *PIG1* putative neutral genomic locus (12). Green arrows denote the 1,182-bp *PpSHR2* promoter sequence. Boxes represent 5' untranslated regions (*PpSHR2* 5'-UTR: black), *PpSHR2* exons (white), a synthetic nucleotide sequence encoding a linker (KGGRADPAFLYKVVITG: purple), *mCitrine* (yellow) (10), the *rbcS* terminator (rbcS-ter: gray) (14), loxP sequences (green) (9), the zeocin resistance cassette (zeo: orange) (16), the *uidA* gene (GUS: light blue) (17), a synthetic nucleotide sequence encoding the SV40 nuclear localization signal (NLS: magenta) (18), *eGFP* (light green), and the blasticidin S deaminase expression cassette (BSD: brown) (19). To generate the PpSHR2pro:PpSHR2-mCitrine and PpSHR2pro:PpSHR2-mCitrine-GUS constructs, the DNA fragment containing 1,182 bp of the *PpSHR2* promoter and the entire coding region until the terminator codon was PCR amplified from wild-type genomic DNA and cloned into pENTR/D-TOPO to generate pENTR-PpSHR2promoter-PpSHR2. The pENTR-PpSHR2promoter-PpSHR2 plasmid was subjected to LR reaction using the destination vectors pTPGmY (OP142292) and pTPGmYG (OP142293) with Gateway LR Clonase II enzyme mix (Thermo Fisher Scientific) to generate the pTPGmY-PpSHR2promoter-PpSHR2 and pTPGmYG-PpSHR2promoter-PpSHR2 plasmids, respectively. To generate the PpSHR2pro:NLS-eGFP-GUS lines, the fragment containing the 1,182-bp promoter fragment and 1,696 bp of 5'-UTR region from *PpSHR2* was PCR amplified from wild-type genomic DNA and cloned into pPIG1-NGGII (20) digested with *Sma*I using In-Fusion HD Cloning Kit (Takara) to generate the pPIG1-PpSHR2pro-NGG plasmid. The generated constructs were digested with *Pme*I for gene targeting and introduced into wild-type plants.

(B) Representative fluorescent images of leaf primordia in PpSHR2pro:PpSHR2-mCitrine#29, PpSHR2pro:PpSHR2-mCitrine-GUS#2, and PpSHR2pro:NLS-eGFP-GUS#28 plants. Optical transverse sections along the yellow dotted lines (upper) are shown in the lower panels. Magenta indicates signals of PpSHR2-mCitrine, PpSHR2-mCitrine-GUS, or NLS-eGFP-GUS fusion proteins. Gametophores of each plant were fixed in 4% (w/v) paraformaldehyde, stained with calcofluor white (shown in Cyan), and then cleared with ClearSee solution (21). (Scale bars, 10 µm.)

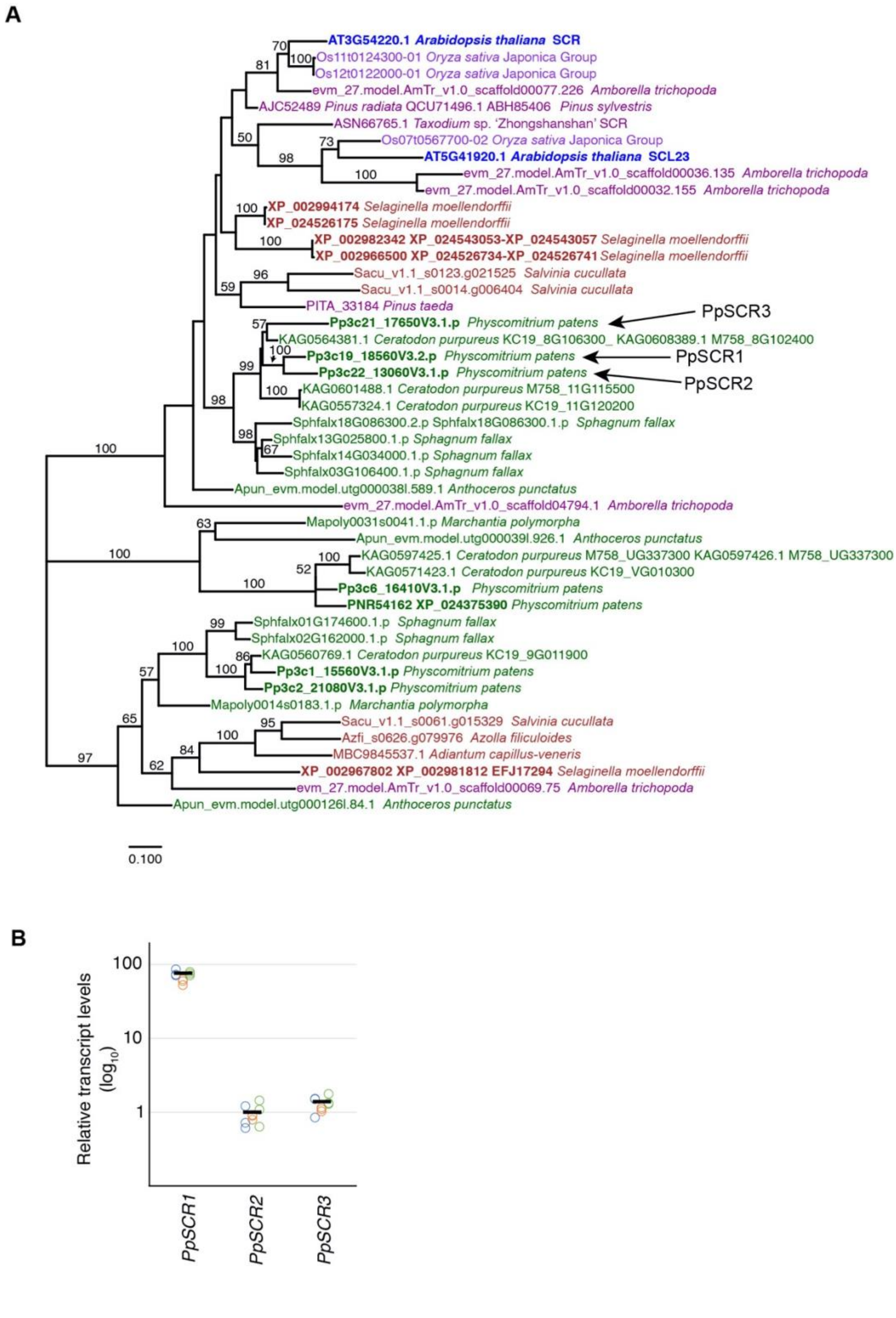
A
AT3G54220.1 Arabidopsis thaliana SCR
Os11t0124300-01 Oryza sativa Japonica Group
Os12t0122000-01 Oryza sativa Japonica Group
evm_27.model.AmTr_v1.0_scaffold00077.226 Amborella trichopoda
AJC52489 Pinus radiata QCU71496.1 ABH85406 Pinus sylvestris
ASN66765.1 Taxodium sp. 'Zhongshanshan' SCR
Os07t0567700-02 Oryza sativa Japonica Group
AT5G41920.1 Arabidopsis thaliana SCL23
evm_27.model.AmTr_v1.0_scaffold00036.135 Amborella trichopoda
evm_27.model.AmTr_v1.0_scaffold00032.155 Amborella trichopoda
XP_002994174 Selaginella moellendorffii
XP_024526175 Selaginella moellendorffii
XP_002982342 XP_024543053-XP_024543057 Selaginella moellendorffii
XP_002966500 XP_024526734-XP_024526741 Selaginella moellendorffii
Sacu_v1.1_s0123.g021525 Salvinia cucullata
Sacu_v1.1_s0014.g006404 Salvinia cucullata
PITA_33184 Pinus taeda
Pp3c21_17650V3.1.p Physcomitrium patens
PpSCR3
KAG0564381.1 Ceratodon purpureus KC19_8G106300_ KAG0608389.1 M758_8G102400
Pp3c19_18560V3.2.p Physcomitrium patens
PpSCR1
Pp3c22_13060V3.1.p Physcomitrium patens
PpSCR2
KAG0601488.1 Ceratodon purpureus M758_11G115500
KAG0557324.1 Ceratodon purpureus KC19_11G120200
Sphfalx18G086300.2.p Sphfalx18G086300.1.p Sphagnum fallax
Sphfalx13G025800.1.p Sphagnum fallax
Sphfalx14G034000.1.p Sphagnum fallax
Sphfalx03G106400.1.p Sphagnum fallax
Apun_evm.model.utg000038l.589.1 Anthoceros punctatus
evm_27.model.AmTr_v1.0_scaffold04794.1 Amborella trichopoda
Mapoly0031s0041.1.p Marchantia polymorpha
Apun_evm.model.utg000039l.926.1 Anthoceros punctatus
KAG0597425.1 Ceratodon purpureus M758_UG337300 KAG0597426.1 M758_UG337300
KAG0571423.1 Ceratodon purpureus KC19_VG010300
Pp3c6_16410V3.1.p Physcomitrium patens
PNR54162 XP_024375390 Physcomitrium patens
Sphfalx01G174600.1.p Sphagnum fallax
Sphfalx02G162000.1.p Sphagnum fallax
KAG0560769.1 Ceratodon purpureus KC19_9G011900
Pp3c1_15560V3.1.p Physcomitrium patens
Pp3c2_21080V3.1.p Physcomitrium patens
Mapoly0014s0183.1.p Marchantia polymorpha
Sacu_v1.1_s0061.g015329 Salvinia cucullata
Azfi_s0626.g079976 Azolla filiculoides
MBC9845537.1 Adiantum capillus-veneris
XP_002967802 XP_002981812 EFJ17294 Selaginella moellendorffii
evm_27.model.AmTr_v1.0_scaffold00069.75 Amborella trichopoda
Apun_evm.model.utg000126l.84.1 Anthoceros punctatus
0.100
B
Relative transcript levels
(log10)
100
10
1
PpSCR1
PpSCR2
PpSCR3

Fig. S6. Phylogeny of SCR in land plants.
(A) Phylogenetic tree of SCR homologs in land plants. The three *Physcomitrium patens* proteins PpSCR1, PpSCR2, and PpSCR3 are indicated with arrows. The maximum likelihood tree under the JTTF model with empirical amino acid frequency was searched with RAxML-HPC, as explained below. The bootstrap probabilities (%) based on 1,000 resamplings are shown on the branches where bootstrap probability was over 50%. The horizontal branch lengths are proportional to the estimated number of substitutions per site, and are drawn to scale. INSD accession numbers or genome-specific identifiers are shown with the species name in which the sequence was present, sometimes followed by the protein name. The OTUs are colored according to the classification: blue, eudicots; blue violet, monocots; dark magenta, other seed plants; brown, other vascular plants (monilophytes and lycophytes); dark green, bryophytes.

SCR homologs were searched from a representative genome collection and nr databases with restriction to the Viridiplantae but excluding angiosperms. The representative genome collection consisted of "Araport11" (*Arabidopsis thaliana* from TAIR), "Atrichopoda1" (*Amborella trichopoda* v 1.0 from Phytozome), "Mpo31" (*Marchantia polymorpha* v 3.1 from Phytozome), "Pita_2_01" (*Pinus taeda* v 2.01, Pita.2_01.peptides.fa.gz on Dec 28 2017 containing 36,732 sequences), "Ppa33" (*Physcomitrella patens* v 3.3 from Phytozome), "rice-IRGSP-1.0" (IRGSP-1.0_protein_2017-08-04.fasta), "Azolla" (*Azolla fillicoides* v1.1 from fernbase [https://www.fernbase.org]), "Salvinia" (*Salvinia* v 1.2 from fernbase), and "Selmo_ncbi" (GCF_000143415.4_v1.0_protein.faa). *Sphagnum fallax* v1.1 (DOE-JGI, http://phytozome.jgi.doe.gov/) was additionally searched at the Phytozome 13 site. The *Anthoceros punctatus* dataset was downloaded from https://www.hornworts.uzh.ch/en/download.html. BLASTP searches were performed with a minimum word size of 2 and allowing up to 1,000 hits of which the top 100 hit target amino acid sequences were retrieved. Thus, the SCR dataset of 100 representative plant + 100 non-angiosperm plants + 51 *Anthoceros punctatus* + 3 *Sphagnum* entries was obtained.

This dataset was aligned with einsi in MAFFT version 7.475 (3). The alignment was imported to Mesquite version 3.61 (4). Redundant entries were not added, as *Selaginella* sequences were contained in both the representative genome set and the non-angiosperm nr dataset. All characters in the alignment were first set as “excluded”. The alignments were reviewed on mesquite, and well-aligned regions were identified and set for “included”. Sequences harboring large deletion(s) in the conserved region were removed. Thus, a 211-entry SCR data matrix was produced and a maximum likelihood tree was constructed. After examining the result, the group derived from a single protein encoded by the gene in the last common ancestor of land plants was estimated, a smaller dataset comprised of the group and two clades of outgroup were chosen (79 entries for SCR), and the alignment was reinvestigated for inclusion/exclusion. The data matrix was saved as a nexus file and subjected to a custom maximum likelihood analysis pipeline involving RAxML-HPC (5). The “included” sites were extracted from the matrix and sequences identical in the included regions were treated as a single OTU during the analysis and reverted to the original names at the final stage. The extracted matrix was converted to PHYLIP format. At first, molecular evolutionary model was chosen using ProteinModelSelection.pl, and in this case, the JTTF (JTT + F) model was selected. JTT stands for amino acid substitution matrix (22) and F stands for using empirical amino acid frequency in the dataset. SEQBOOT (6) was used to prepare 1,000 resampling sets. The original and bootstrap replicates were individually processed with the “-f a -# 100” option in RAxML. The model was specified with “-m PROTGAMMALGF” or “-m PROTGAMMAJTTF” according to the chosen model. Random number seeds -x and -p option were taken from the operating system with /dev/urandom. After all jobs had finished, the resulting trees were recovered and the bootstrap frequency was calculated using CONSENSE (6). To force the topology to be the same as that of the tree obtained from the original dataset, the original tree was amplified 1,000 times and combined with the bootstrap results; after consensus calculation, 1,000 was subtracted from the result.
(B) Quantification of relative *PpSCR1*, *PpSCR2,* and *PpSCR3* transcript levels in gametophores. The value of *PpSCR2* transcripts was set to 1.0. Three biological replicates (blue, orange, and green plots) with three technical replicates were performed. The horizontal bars indicate the means.

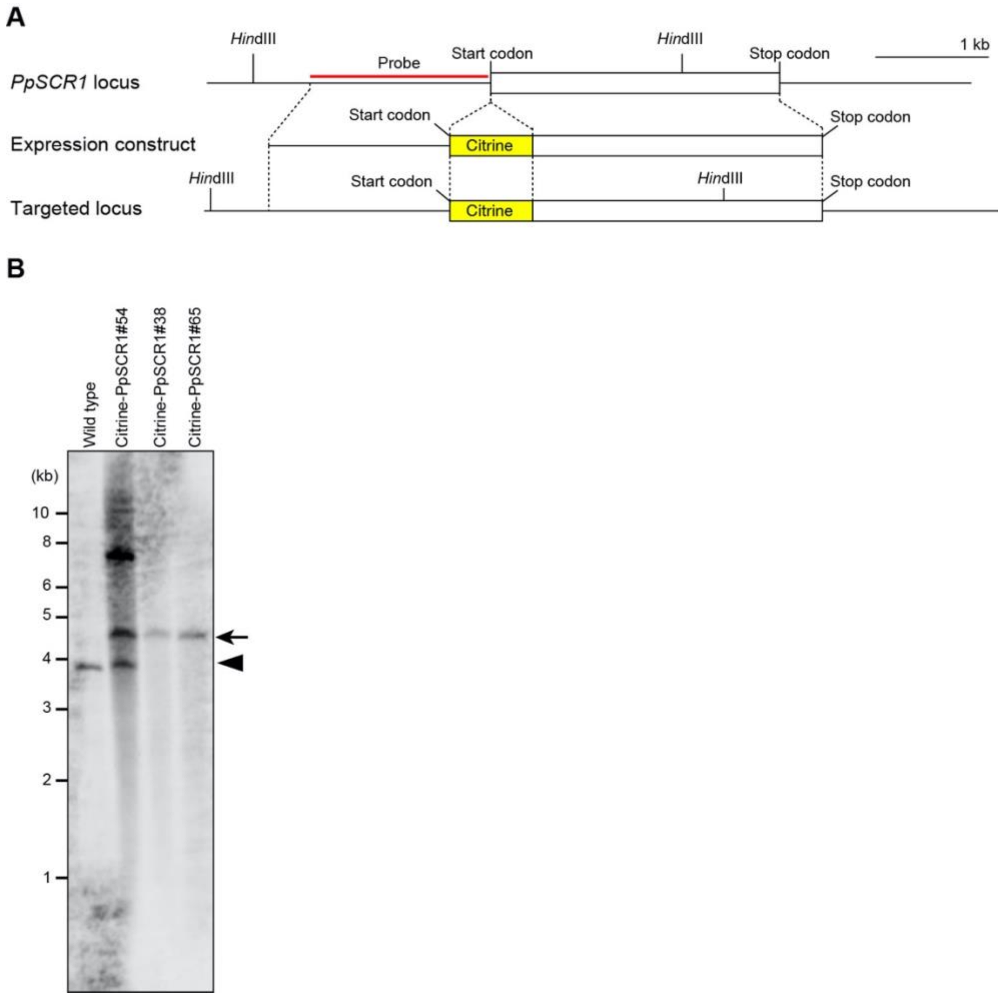

Fig. S7. Construction of Citrine-PpSCR1 plants.
(A) Schematic diagram of the construct targeting the *PpSCR1* locus. White and yellow boxes represent *PpSCR1* exons and *Citrine* (11), respectively. Probes used in (B) are indicated as red horizontal lines. To insert the *Citrine* gene in-frame with the *PpSCR1* coding sequence, a genomic DNA fragment spanning from the start codon to the stop codon of *PpSCR1* was inserted into the *Bsr*GI site of the pCit-aphIV plasmid (LC703380), resulting in the generation of the in-frame *Citrine-PpSCR1* fusion. The *Citrine-PpSCR1* fragment was then PCR amplified and inserted into the pENTR/D-TOPO vector (Thermo Fisher Scientific) to generate the pENTR:Citrine-PpSCR1 plasmid. A genomic DNA fragment including a partial sequence of the *PpSCR1* promoter and the 5' untranslated region was amplified from wild-type genomic DNA and inserted at the 5' end of *Citrine* in the plasmid pENTR:Citrine-PpSCR1. The generated construct was digested with suitable restriction enzymes for gene targeting and introduced into wild-type plants.
(B) DNA gel blot analyses of targeted plants with the probes indicated in (A). Genomic DNA of wild-type and Citrine-PpSCR1 (#54, #38, and #65) plants was digested with *Hin*dIII. The arrowhead and arrow indicate DNA fragments specific to wild-type and Citrine-PpSCR1 plants, respectively.

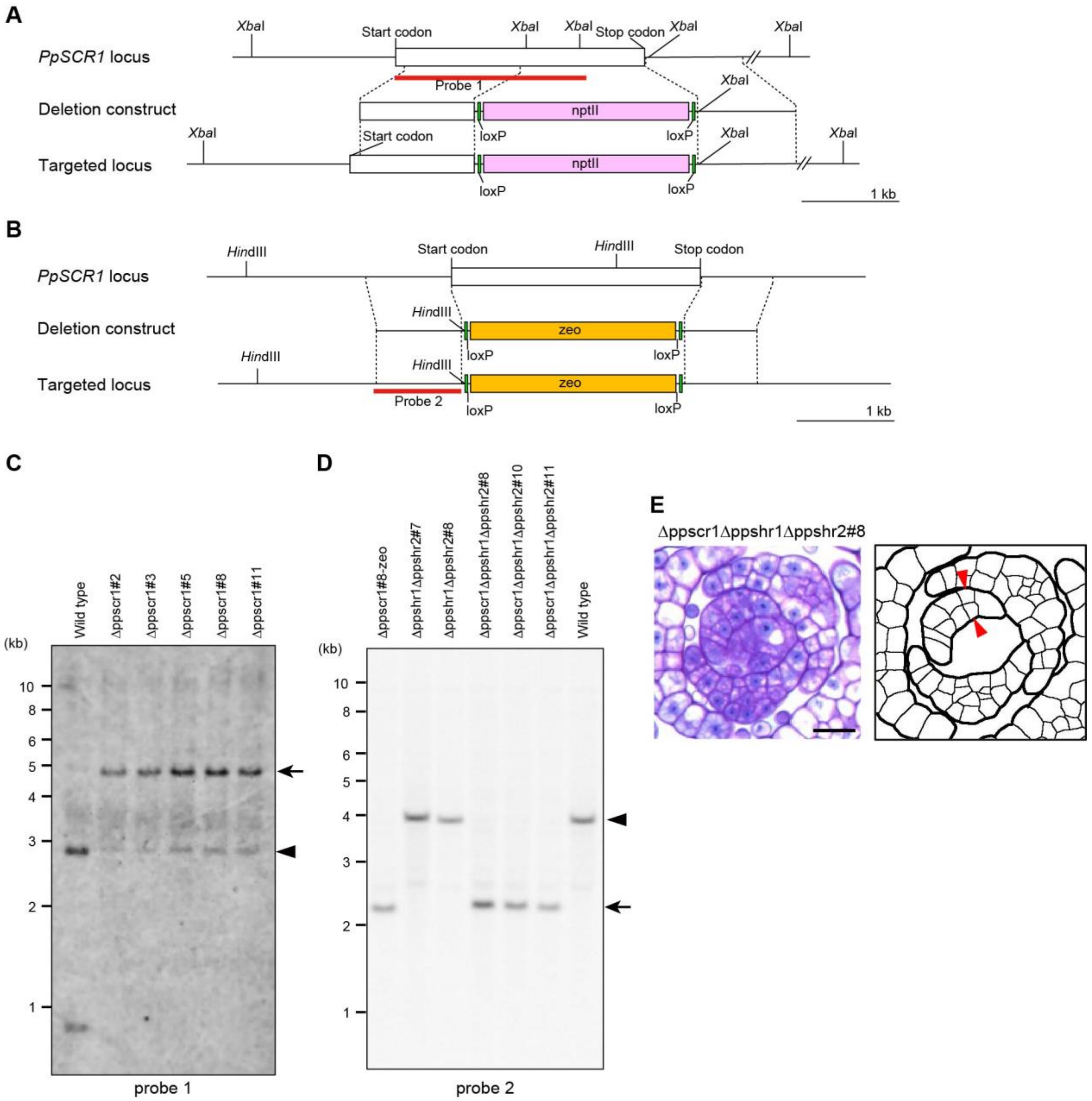

Fig. S8. Construction of Δppscr1 and Δppscr1Δppshr1Δppshr2 plants.

(A and B) Schematic diagram of the construct targeting the *PpSCR1* locus. White boxes represent *PpSCR1* exons. Magenta, orange, and green boxes denote the neomycin phosphotransferase II expression cassette (nptII) (7), the zeocin resistance cassette (zeo) (16), and loxP sequences, respectively. Probes used in (C and D) are indicated as red horizontal lines. To delete *PpSCR1*, two types of deletion constructs, Δppscr1-nptII and Δppscr1-zeo, were generated. In the plasmid Δppscr1-nptII (A), the coding region encoding the GRAS domain of the *PpSCR1* gene was deleted. Genomic fragments containing the partial coding sequence and 3' flanking regions of the *PpSCR1* gene were amplified and inserted into the 5' and 3' ends, respectively, of the nptII expression cassette of plasmid pTN182. In the plasmid Δppscr1-zeo (B), the full-length *PpSCR1* gene was deleted. Genomic fragments containing the 5' and 3' flanking regions of the gene were amplified and inserted into the 5' and 3' ends, respectively, of the zeo expression cassette of the plasmid p35S-loxP-Zeo (AB540628). The two constructs

were digested with suitable restriction enzymes for gene targeting and introduced into wild-type plants to generate ∆ppscr1 (C) and ∆ppscr1-zeo (D) single deletion mutants.
(C) DNA gel blot analysis of ∆ppscr1 plants. Genomic DNA of wild-type and ∆ppscr1 (#2, #3, #5, #8, and #11) plants was digested with *Xba*I.
(D) DNA gel blot analysis of ∆ppscr1∆ppshr1∆ppshr2 plants. Genomic DNA of wild-type, ∆ppscr1-zeo#8, ∆ppshr1∆ppshr2 (#7 and #8) and ∆ppscr1∆ppshr1∆ppshr2 (#8, #10, and #11) plants was digested with *Hin*dIII. To generate ∆ppscr1∆ppshr1∆ppshr2 triple deletion mutants, the ∆ppscr1-zeo plasmid (B) was introduced into the ∆ppshr1∆ppshr2#7 double mutant plant.
(E) Representative cross-section of a gametophore apex of ∆ppscr1∆ppshr1∆ppshr2#8 line. A schematic illustration is shown to the right. Each leaf is surrounded by a thick line. Red arrowheads indicate most medial lateral (mml) daughter cells after periclinal cell division. (Scale bar, 20 µm.)

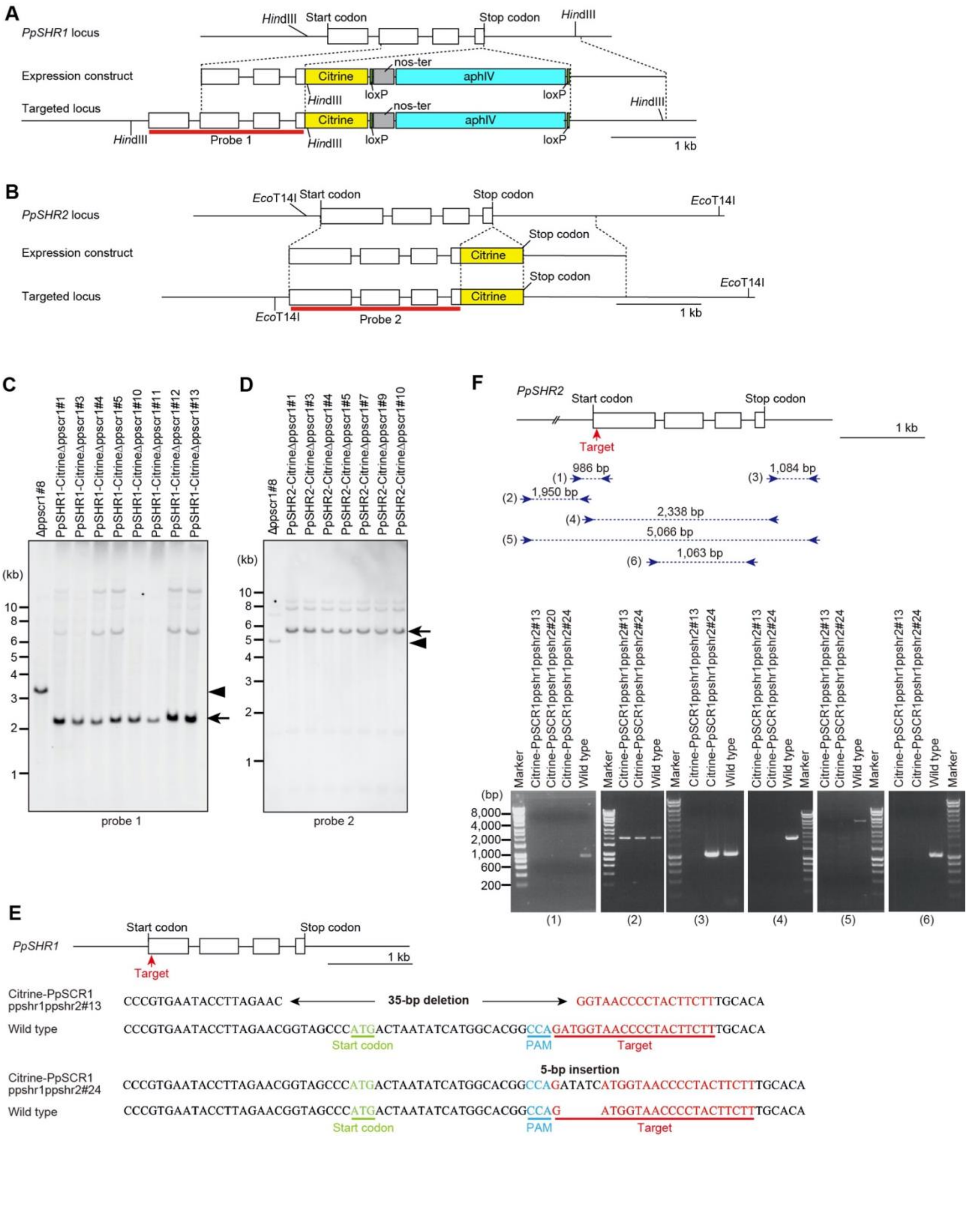
A
PpSHR1 locus
HindIII
Start codon
Stop codon
Expression construct
Citrine
nos-ter
aphIV
loxP
Targeted locus
Probe 1
1 kb
B
PpSHR2 locus
EcoT14I
Expression construct
Targeted locus
Probe 2
C
Δppscr1#8
PpSHR1-CitrineΔppscr1#1
PpSHR1-CitrineΔppscr1#3
PpSHR1-CitrineΔppscr1#4
PpSHR1-CitrineΔppscr1#5
PpSHR1-CitrineΔppscr1#10
PpSHR1-CitrineΔppscr1#11
PpSHR1-CitrineΔppscr1#12
PpSHR1-CitrineΔppscr1#13
(kb)
probe 1
D
PpSHR2-CitrineΔppscr1#1
PpSHR2-CitrineΔppscr1#3
PpSHR2-CitrineΔppscr1#4
PpSHR2-CitrineΔppscr1#5
PpSHR2-CitrineΔppscr1#7
PpSHR2-CitrineΔppscr1#9
PpSHR2-CitrineΔppscr1#10
probe 2
F
PpSHR2
Target
986 bp
1,084 bp
1,950 bp
2,338 bp
5,066 bp
1,063 bp
Marker
Citrine-PpSCR1ppshr1ppshr2#13
Citrine-PpSCR1ppshr1ppshr2#20
Citrine-PpSCR1ppshr1ppshr2#24
Wild type
(bp)
8,000
4,000
2,000
1,000
600
200
E
PpSHR1
Citrine-PpSCR1 ppshr1ppshr2#13
CCCGTGAATACCTTAGAAC 35-bp deletion GGTAACCCCTACTTCTTTGCACA
Wild type
CCCGTGAATACCTTAGAACGGTAGCCCATGACTAATATCATGGCACGGCCAGATGGTAACCCCTACTTCTTTGCACA
Start codon
PAM
Target
Citrine-PpSCR1 ppshr1ppshr2#24
5-bp insertion
CCCGTGAATACCTTAGAACGGTAGCCCATGACTAATATCATGGCACGGCCAGATATCATGGTAACCCCTACTTCTTTGCACA
Wild type
CCCGTGAATACCTTAGAACGGTAGCCCATGACTAATATCATGGCACGGCCAG ATGGTAACCCCTACTTCTTTGCACA

Fig. S9. Construction of PpSHR1-CitrineΔppscr1, PpSHR2-CitrineΔppscr1, and Citrine-PpSCR1ppshr1ppshr2 plants.

(A and B) Schematic diagrams of the targeting strategy for the *PpSHR1* (A) and *PpSHR2* (B) loci. White boxes represent exons. Yellow, cyan, gray, and green boxes denote *Citrine* (11), the aminoglycoside phosphotransferase IV expression cassette (aphIV) (8), the *nopaline synthase* terminator (nos-ter) (7), and loxP sequences (9), respectively. Probes used in (C and D) are indicated as red horizontal lines. To insert *Citrine* in-frame with the *PpSHR1* coding sequence, a *PpSHR1* genomic DNA fragment extending from the middle to the last codon and the 3' flanking region were separately PCR amplified from wild-type genomic DNA. The two amplified fragments and the Citrine expression construct including Citrine and the aphIV expression cassette (8) from the pCit-aphIV plasmid were precisely inserted into the pBluescriptII plasmid (Agilent). The generated construct was digested with suitable restriction enzymes for gene targeting and introduced into wild-type plants.
(C and D) DNA gel blot analysis of targeted lines. (C) Genomic DNA of Δppscr1#8 and PpSHR1-CitrineΔppscr1 (#1, #3, #4, #5, #10, #11, #12, and #13) plants was digested with *Hin*dIII*.* (D) Genomic DNA of Δppscr1#8 and PpSHR2-CitrineΔppscr1 (#1, #3, #4, #5, #7, #9, and #10) plants was digested with *Eco*T14I. The Δppscr1#8 was used to generate PpSHR1-CitrineΔppscr1 and PpSHR2-CitrineΔppscr1 plants. Arrowheads and arrows indicate DNA fragments specific to Δppscr1#8 and PpSHR1-CitrineΔppscr1 (C), or PpSHR2-CitrineΔppscr1 (D), respectively.
(E and F) Construction of Citrine-PpSCR1ppshr1ppshr2 plants using the CRISPR/Cas9 system (23, 24). To introduce mutations in the *PpSHR1* and *PpSHR2* genes by the CRISPR/CAS9 system, primers were designed by CRISPRdirect (http://cridpr.dbcls.jp) to produce single guide RNAs (sgRNAs) targeting *PpSHR1* or *PpSHR2.* The primer sequences are listed in Table S1. The primers for *PpSHR1* and *PpSHR2* were annealed and cloned at the *Bsa*I site of the sgRNA expression plasmid pPpU6-sgRNA (LC494193) to generate pPpU6-*PpSHR1*-sgRNA and pPpU6-*PpSHR2*-sgRNA, respectively. Schematic diagrams illustrating the design of the sgRNA targeting *PpSHR1* (E) or *PpSHR2* (F) are shown. White boxes denote exons. The two plasmids were simultaneously introduced into Citrine-PpSCR1#38 plants. The resulting mutant plants were screened for deletions or insertions at the *PpSHR1* and *PpSHR2* loci using Sanger sequencing and genomic PCR analyses. Sequences of the *PpSHR1* locus in the wild type and mutants are shown at the bottom in (E). Light green, light blue, and red letters denote the start codon of *PpSHR1*, the protospacer adjacent motif (PAM), and the sgRNA target sequences, respectively. Genomic DNA of wild type and Citrine-PpSCR1ppshr1ppshr2 (#13, #20, #24) plants was purified and subjected to Sanger sequencing (E) and genomic PCR analyses with six primer pairs (1 to 6) shown as arrows (F). The primer sequences for genomic PCR to evaluate the *PpSHR2* locus are described in Table S2.

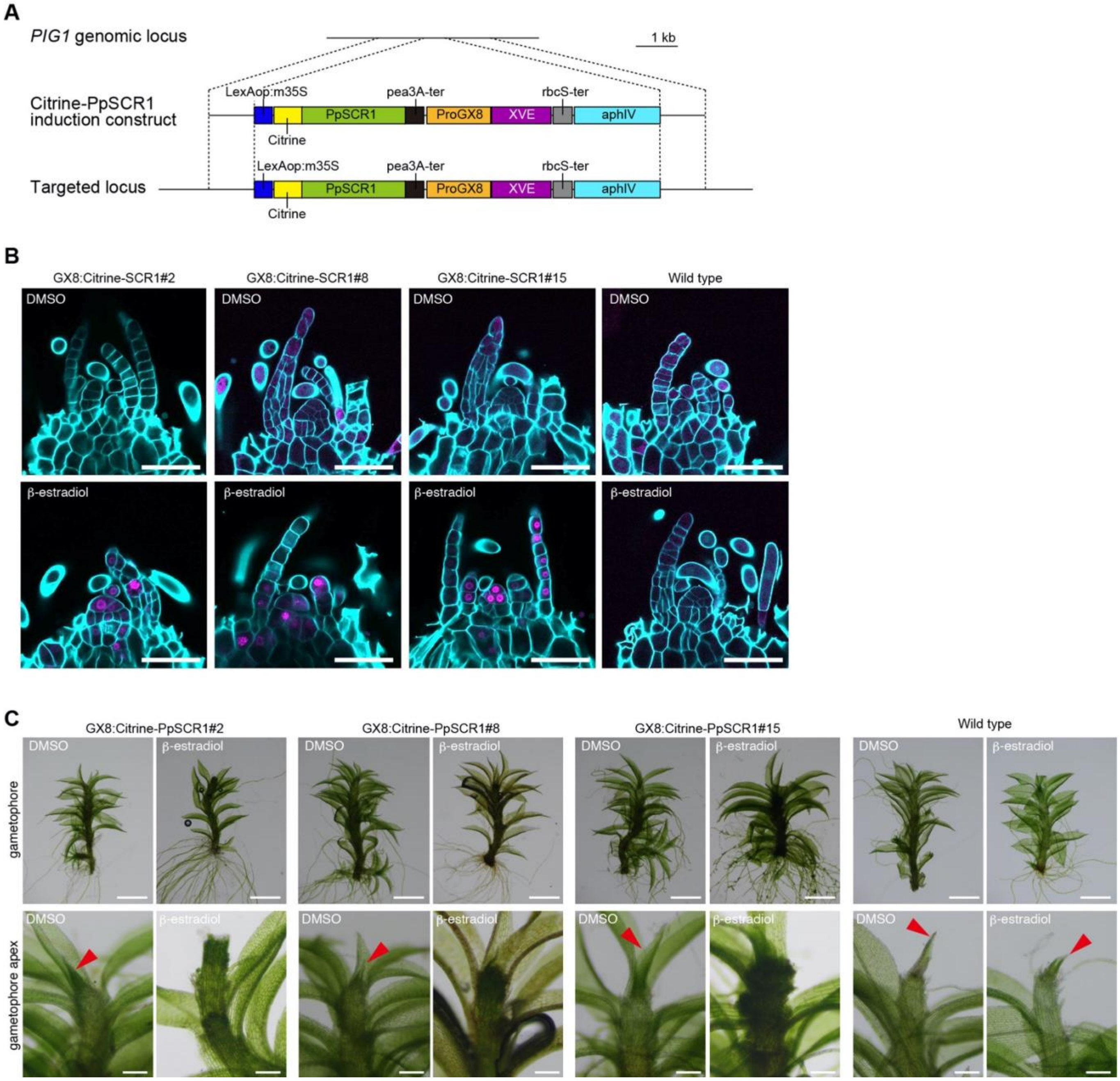

Fig. S10. Construction of GX8:Citrine-PpSCR1 plants and Citrine-PpSCR1 accumulation pattern in the shoot apex.

(A) Schematic diagram showing the insertion strategy of the *Citrine-PpSCR1* induction construct into the *PIG1* putative neutral genomic locus (12). The connected DNA fragment of the *LexA* operator and minimal *35S* promoter (blue: LexAop:m35S) (13), *Citrine* (yellow) (11), *PpSCR1* (green), the pea *rbcS3A* terminator (black: pea3A-ter) (14), the putative *ProGX8* promoter (orange) (12), a DNA fragment encoding an XVE fusion protein derived from pER8 (purple) (13), the *rbcS* terminator (gray, rbcS-ter) (14), and the aminoglycoside phosphotransferase IV expression cassette (aphIV： cyan) (8) are shown in different colors. To generate the Citrine-PpSCR1 expression construct, the pENTR:Citrine-PpSCR1 plasmid (Fig. *S7A*) was subjected to LR reaction using the destination vector pPGX8 (AB537482) (12) to generate the GX8:Citrine-PpSCR1 plasmid. The

generated construct was digested with the restriction enzyme *Pme*I for gene targeting and introduced into wild-type plants.
(B) Accumulation pattern of Citrine-PpSCR1 protein in gametophore apices from GX8:Citrine-PpSCR1 plants. Young gametophores of each line were cultivated in liquid BCD medium with or without 1 µM β-estradiol for three days and stained with calcofluor white. Optical longitudinal sections of apical parts of gametophores with leaf primordia are indicated. Magenta and cyan indicate the signals of Citrine-PpSCR1 and calcofluor, respectively. (Scale bar, 50 µm.)
(C) Gametophores (upper) and gametophore apices (lower) of the GX8:Citrine-PpSCR1 and wild-type plants cultivated with or without 1 µM β-estradiol for 31 days. To observe gametophore apices, leaves surrounding gametophore apices were removed. Magnified images of these gametophore apices are shown in the lower panels. Red arrows indicate young leaves. Note that young leaves did not grown in the GX8:Citrine-PpSCR1 plants cultivated with 1 µM β-estradiol. (Scale bars, 1 mm in upper panels, 200 µm in lower panels.)

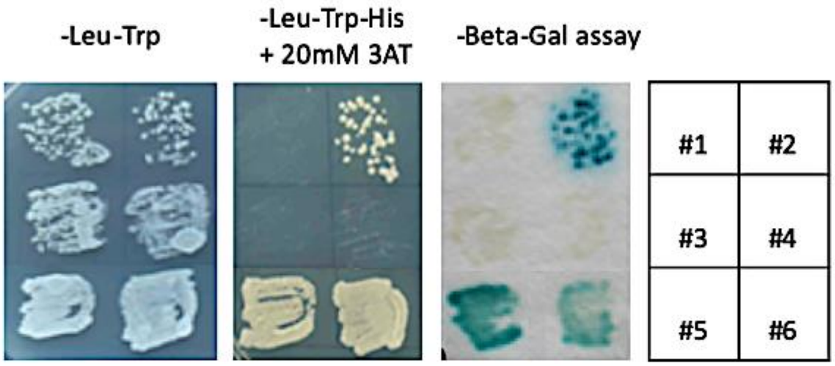

Fig. S11. Protein-protein interaction of PpSHRs and PpSCR1 in yeast cells.
1. GAL4-DNA Binding Domain (BD)+GAL4-Activation Domain (AD) as a negative control, 2. BD-*Arabidopsis thaliana* SHR (AtSHR)+AD-AtSHR as a positive control, 3. BD-PpSHR1+AD, 4. BD-PpSHR2+AD, 5. BD-PpSHR1+AD-PpSCR1, 6. BD-PpSHR2+AD-PpSCR1. Yeast cells (1 to 6) were streaked on the synthetic complete (SC) medium without leucine and tryptophane (SC-Leu-Trp; left) or the SC medium without leucine, tryptophane, and histidine (SC-Leu-Trp-His; middle). The latter contains 20 mM 3-amino-1,2,4-triazole (3AT) to suppress the auto-transcriptional activity of PpSHR1, PpSHR2, and PpSCR1 used as baits. Beta-galactosidase assay was also preformed (right).

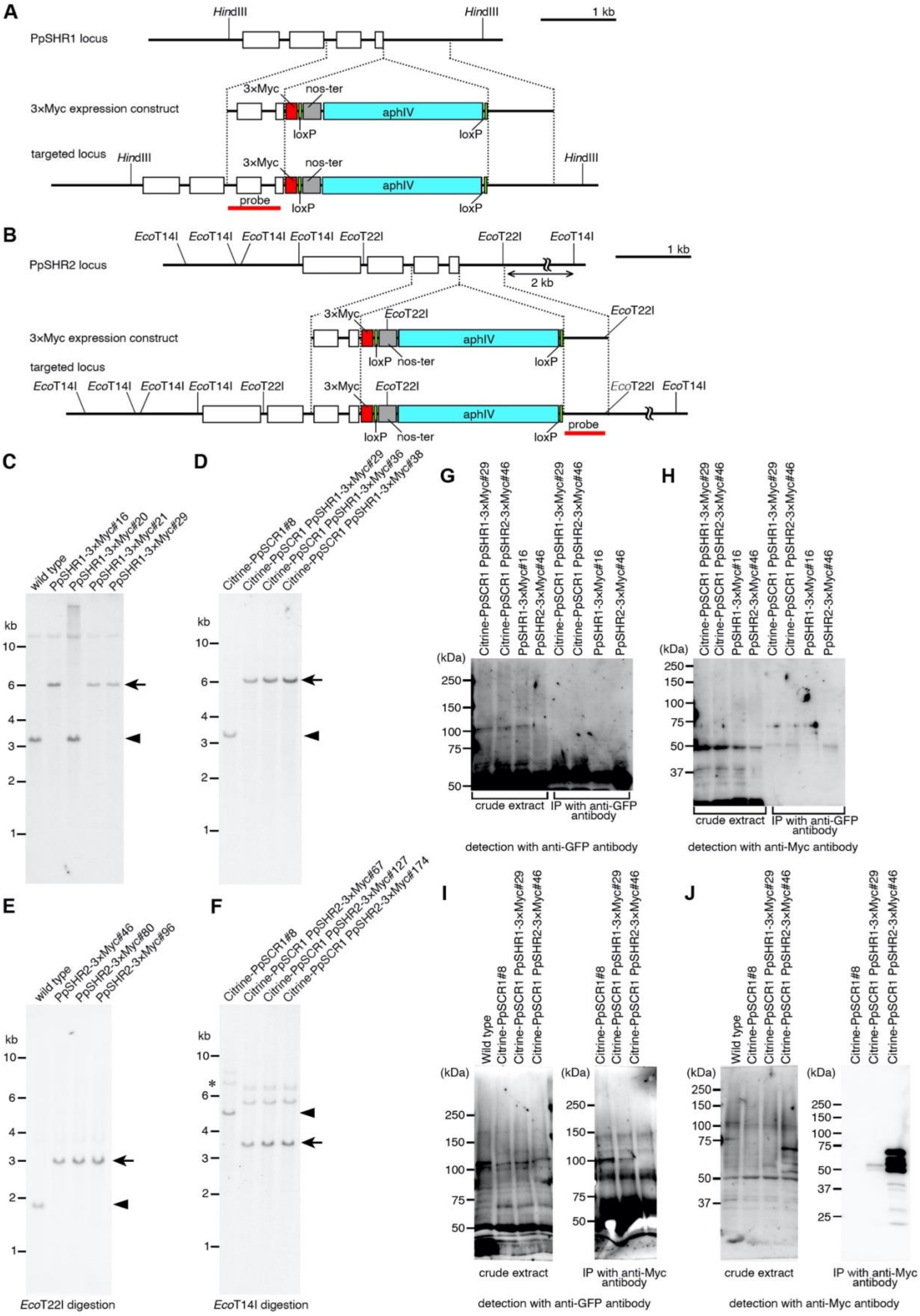

A
PpSHR1 locus
HindIII
1 kb
3×Myc expression construct
3×Myc
nos-ter
aphIV
loxP
targeted locus
probe
B
PpSHR2 locus
EcoT14I
EcoT22I
2 kb
C
wild type
PpSHR1-3×Myc#16
PpSHR1-3×Myc#20
PpSHR1-3×Myc#21
PpSHR1-3×Myc#29
kb
D
Citrine-PpSCR1#8
Citrine-PpSCR1 PpSHR1-3×Myc#29
Citrine-PpSCR1 PpSHR1-3×Myc#36
Citrine-PpSCR1 PpSHR1-3×Myc#38
E
PpSHR2-3×Myc#46
PpSHR2-3×Myc#80
PpSHR2-3×Myc#96
EcoT22I digestion
F
Citrine-PpSCR1 PpSHR2-3×Myc#67
Citrine-PpSCR1 PpSHR2-3×Myc#127
Citrine-PpSCR1 PpSHR2-3×Myc#174
EcoT14I digestion
G
Citrine-PpSCR1 PpSHR2-3×Myc#46
PpSHR1-3×Myc#16
PpSHR2-3×Myc#46
(kDa)
crude extract
IP with anti-GFP antibody
detection with anti-GFP antibody
H
detection with anti-Myc antibody
I
Wild type
IP with anti-Myc antibody
J

Fig. S12. Analysis of protein-protein interaction between PpSCR and PpSHR in gametophores.
(A and B) Schematic diagram of the constructs used to add Myc tags to the C-terminal ends of PpSHR1 (A) and PpSHR2 (B) at the endogenous loci. White boxes represent exons. A triple Myc-tag (3×Myc: red), loxP (green) (9), the nopaline synthase terminator (gray: nos-ter) (7), and the aminoglycoside phosphotransferase IV expression cassette (aphIV: cyan) (8) are shown in different colors. Probes used in (C-F) are indicated as horizontal red lines. To insert the DNA fragments encoding a triple Myc-tag (3×Myc) at the *PpSHR1* and *PpSHR2* loci in wild-type and Citrine-PpSCR1#38 plants, a genomic DNA fragment of each gene from the middle to the last codon of the coding sequence was PCR amplified from wild-type genomic DNA. Primers used for plasmid construction are listed in Table S1. The amplified fragments were inserted in-frame into the 5' end of the DNA fragments encoding 3×Myc in the p3×Myc-aph4 plasmid (LC101764), thereby creating in-frame fusion genes. Genomic fragments containing the 3' flanking region of each gene were inserted into the 3' region of the aphIV expression cassette of the plasmids. The generated constructs were digested with suitable restriction enzymes for gene targeting.
(C and D) Genomic DNA of wild-type, PpSHR1-3×Myc (#16, #20, #21, and #29), and Citrine-PpSCR1 PpSHR1-3×Myc (#29, #36, and #38) plants was digested with *Hin*dIII*.* Arrowheads and arrows indicate DNA fragments specific to wild-type (C) or Citrine-PpSCR1#8 (D) and PpSHR1-3×Myc (C) or Citrine-PpSCR1 PpSHR1-3×Myc (D) plants, respectively.
(E and F) Genomic DNA of wild type, PpSHR2-3×Myc (#46, #80, and #96), and Citrine-PpSCR1 PpSHR2-3×Myc (#67, #127, and #174) plants was digested with *Eco*T22I (E) or *Eco*T14I (F)*.* Arrowheads and arrows indicate DNA fragments specific to wild type (E) or Citrine-PpSCR1 (F) and PpSHR2-3×Myc (E) or Citrine-PpSCR1 PpSHR2-3×Myc (F) plants.
(**G to J**) Citrine-PpSCR1 does not co-immunoprecipitate with Myc-tagged PpSHR1 or PpSHR2. (G and H) Crude extracts were prepared from four-week-old gametophores of Citrine-PpSCR1 PpSHR1-3×Myc#29, Citrine-PpSCR1 PpSHR2-3×Myc#46, PpSHR1-3×Myc#16, and PpSHR2-3×Myc#46 plants and subjected to immunoprecipitation with anti-GFP antibody. Crude extracts and immunoprecipitates (IP) were analyzed with anti-GFP (G) and anti-Myc (H) antibodies. (I and J) Crude extracts were prepared from four-week-old gametophores of Citrine-PpSCR1#8, Citrine-PpSCR1 PpSHR1-3×Myc#29, and Citrine-PpSCR1 PpSHR2-3×Myc#46 plants and subjected to immunoprecipitation with anti-Myc antibody. Crude extracts and immunoprecipitates (IP) were analyzed with anti-GFP (I) and anti-Myc (J) antibodies. Crude extracts from wild type were used as a control to evaluate the band specific to Citrine-PpSCR1. No band specific to Citrine-PpSCR1 protein was detected with anti-GFP antibody in crude extracts from Citrine-PpSCR1#8, Citrine-PpSCR1 PpSHR1-3×Myc#29, or Citrine-PpSCR1 PpSHR2-3×Myc#46 or in their immunoprecipitates.

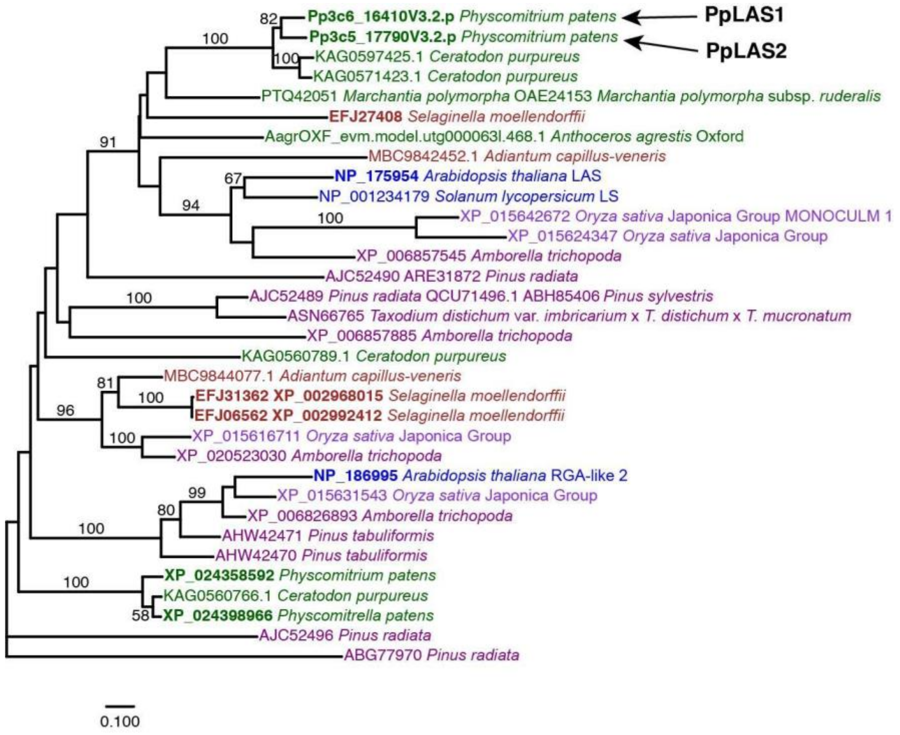

Fig. S13. Phylogeny of LAS in land plants.
Maximum likelihood tree of LAS-related proteins. The two *Physcomitrium patens* proteins PpLAS1 and PpLAS2 are indicated with arrows. Phylogenetic analysis was performed using amino acid sequences found by BLAST searches using tomato (*Solanum lycopersicum*) LS (Lateral suppressor) as a query. The maximum likelihood tree under the LG model (1) with empirical amino acid frequency was searched with RAxML-HPC. The horizontal branch lengths are proportional to the estimated number of substitutions / site, and are drawn to scale. Bootstrap values over 50% are shown on each branch. INSD accession numbers or genome-specific identifiers are shown with the species name in which the sequence was present, sometimes followed by the protein name. The OTUs are colored according to the classification: blue, eudicots; blue violet, monocots; dark magenta, other seed plants; brown, other vascular plants (monilophytes and lycophytes); dark green, bryophytes.

LAS homologs were searched at NCBI using the tomato LS amino acid sequence NP_001234179.1 as a query as of April 7, 2021. The search was divided in three targets: 1) Streptophyta (taxid:35493) but excluding: Spermatophyta (taxid:58024), 2) Spermatophyta but not angiosperms, and 3) refseq protein of Arabidopsis, rice (*Oryza sativa*), and *Amborella trichopoda*. Minimum word size was set to 2, with a maximum hit list size of 1,000. These searches recovered 20, 10, and 10 sequences, respectively. The *PpLAS* coding sequences were subjected to BLASTX search against *Physcomitrium patens* proteins and revealed that *PpLAS1* encodes a protein identical to XP_024377672.1, whereas *PpLAS2* encodes a large portion identical of XP_024375390.1, but with a different N-terminal structure. PHYPA_007838 was also effectively identical to *PpLAS2*. Therefore, these three entries were removed from the dataset. Hornwort (25, 26) and Zygnematales (27) homologs were searched on their respective recently published sets. Each of the four hornwort protein sets contained one high similarity gproteinene with 288-294 bits score of similarity, while the next one dropped to 249 bits. Thus, the top hit from *Anthoceros agrestis* Oxford isolate was retained. The hit to the two Zygnematales algae were limited to 230 bits and no sequence was taken.

The dataset was aligned with einsi in MAFFT version 7.475 (3). The alignment was imported to Mesquite version 3.61. (4). Redundant entries were not added, as *Selaginella* sequences were contained in both the representative genome set and the non-angiosperm nr dataset. All characters in the alignment were first set as “excluded”. The alignments were reviewed on Mesquite and well-aligned regions were identified and set to “included”. Sequences having large deletion(s) in the conserved region were removed. For the LAS dataset, Adiantum sequence MBC9838938.1 was removed due to a lack of otherwise conserved “RF” sequence. After “excluding” all characters, only well-conserved blocks were included to result in 247 amino acid sites. Each data

matrix was saved as a nexus file and subjected to a custom maximum likelihood analysis pipeline involving RAxML-HPC (5). The “included” sites were extracted from the matrix and sequences identical in the included regions were treated as a single OTU during the analysis and reverted to the original names at the final stage. The extracted matrix was converted to PHYLIP format. At first, a molecular evolutionary model was chosen using ProteinModelSelection.pl, and in this case, LGF (LG+F) was chosen, where LG stands for the amino acid substitution matrix by Le and Gascuel (1) and F stands for using empirical amino acid frequency in the dataset. SEQBOOT (6) was used to prepare 1,000 resampling sets. The original and bootstrap replicates were individually processed with the “-f a -# 100” option in RAxML. The model was specified with “-m PROTGAMMALGF” according to the chosen model. Random number seeds -x and -p options were taken from the operating system with /dev/urandom. After all jobs finished, the resulting trees were recovered and the bootstrap frequency was calculated using CONSENSE (6). To force the topology to be the same as the tree obtained from the original dataset, the original tree was amplified 1,000 times and combined with the bootstrap results; after consensus calculation, 1,000 was subtracted from the result.

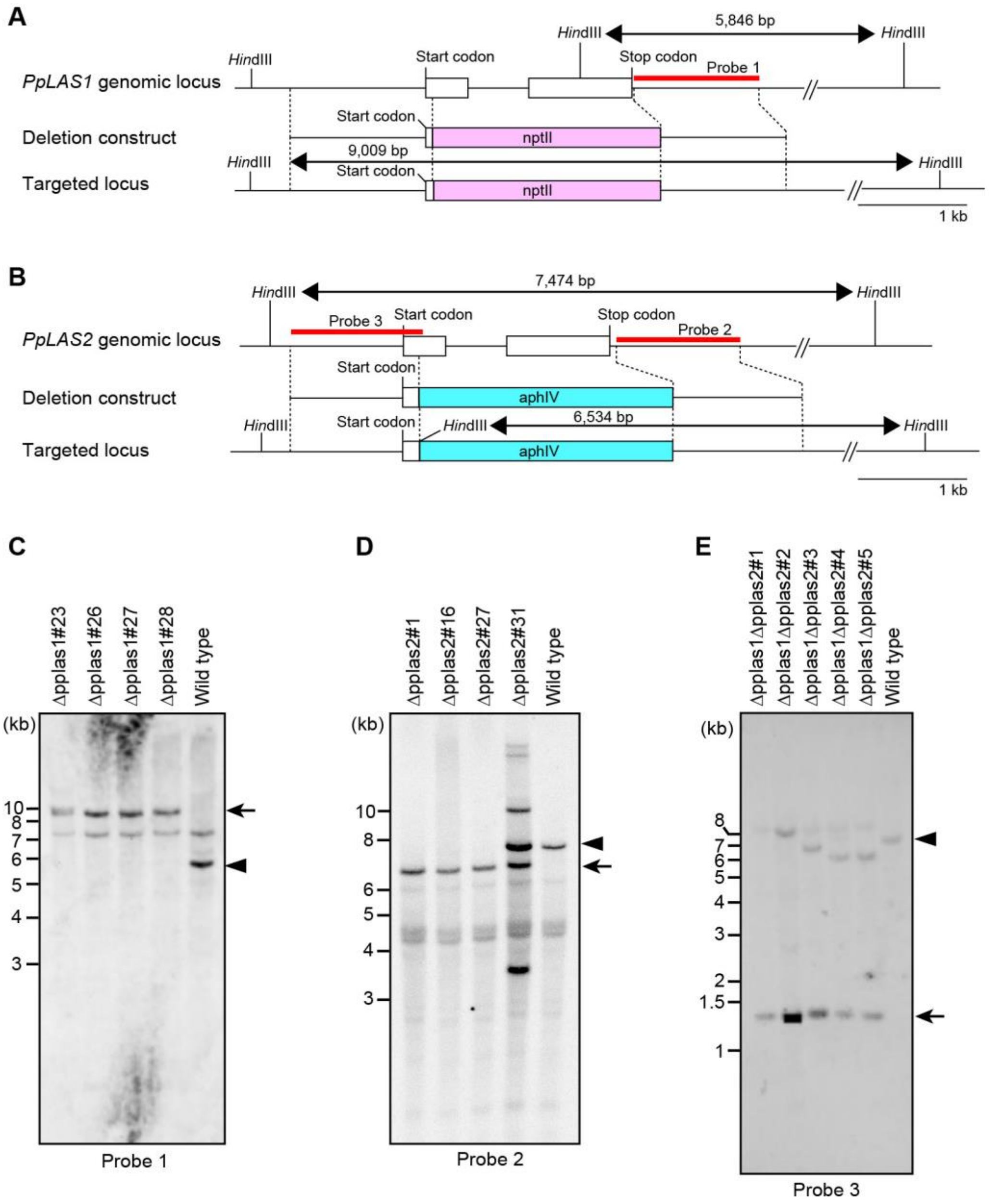

Fig. S14. Construction of ∆pplas1, ∆pplas2, and ∆pplas1∆pplas2 plants.
(A and B) Schematics of construct targeting *PpLAS1* and *PpLAS2* loci. White boxes represent exons. Pink and cyan boxes denote the neomycin phosphotransferase II expression cassette (nptII) (7) and the aminoglycoside phosphotransferase IV expression cassette (aphIV) (8), respectively. Probes used in (C-E) are indicated as red horizontal lines. To assemble the *PpLAS1* and *PpLAS2* gene deletion constructs, genomic fragments containing the 5' flanking region and a partial coding sequence of each *PpLAS* gene were inserted into the 5' end of the nptII expression cassette of plasmid pTN182 for ∆pplas1 and of the aphIV expression cassette of plasmid pTN186 for ∆pplas2. The 3' flanking region of each *PpLAS* gene was inserted into the 3' region of the nptII and the aphIV expression cassettes of the resulting plasmids. The generated constructs were digested with suitable restriction enzymes for gene targeting and introduced into wild-type plants to generate ∆pplas1 (C) and ∆pplas2 (D) single deletion mutants.

(C) DNA gel blot analysis of ∆pplas1 plants. Genomic DNA of wild-type and ∆pplas1 (#23, #26, #27, and #28) plants was digested with *Hin*dIII*.* The arrowhead and arrow indicate DNA fragments specific to wild-type and ∆pplas1 plants, respectively.
(D) DNA gel blot analysis of ∆pplas2 plants. Genomic DNA of wild-type and ∆pplas2 (#1, #16, #27, and #31) plants was digested with *Hin*dIII. The arrowhead and an arrow indicate DNA fragments specific to wild-type and ∆pplas2 plants, respectively.
(E) DNA gel blot analysis of ∆pplas1∆pplas2 plants. Genomic DNA of wild-type and ∆pplas1∆pplas2 (#1, #2, #3, #4, and #5) plants was digested with *Hin*dIII. The arrowhead and arrow indicate DNA fragments specific to wild-type and ∆pplas1∆pplas2 plants, respectively. The *PpLAS2* deletion construct was introduced into ∆pplas1#23 and ∆pplas1#26 plants to generate ∆pplas1∆pplas2#1 and ∆pplas1∆pplas2#2 to #5 double deletion plants, respectively. The circular plasmid pTN75 (AB542060), which contains a Cre recombinase expression cassette, was transiently expressed in the ∆pplas1∆pplas2#1 double deletion mutant plant by polyethylene glycol (PEG)-mediated transformation. The nptII and the aphIV expression cassettes located between the two loxP sites were removed by the Cre recombinase. Loss of the antibiotic expression cassettes was confirmed on the basis of failure to grow on medium containing G418 or hygromycin. The newly generated plants were renamed as ∆pplas1∆pplas2-mf.

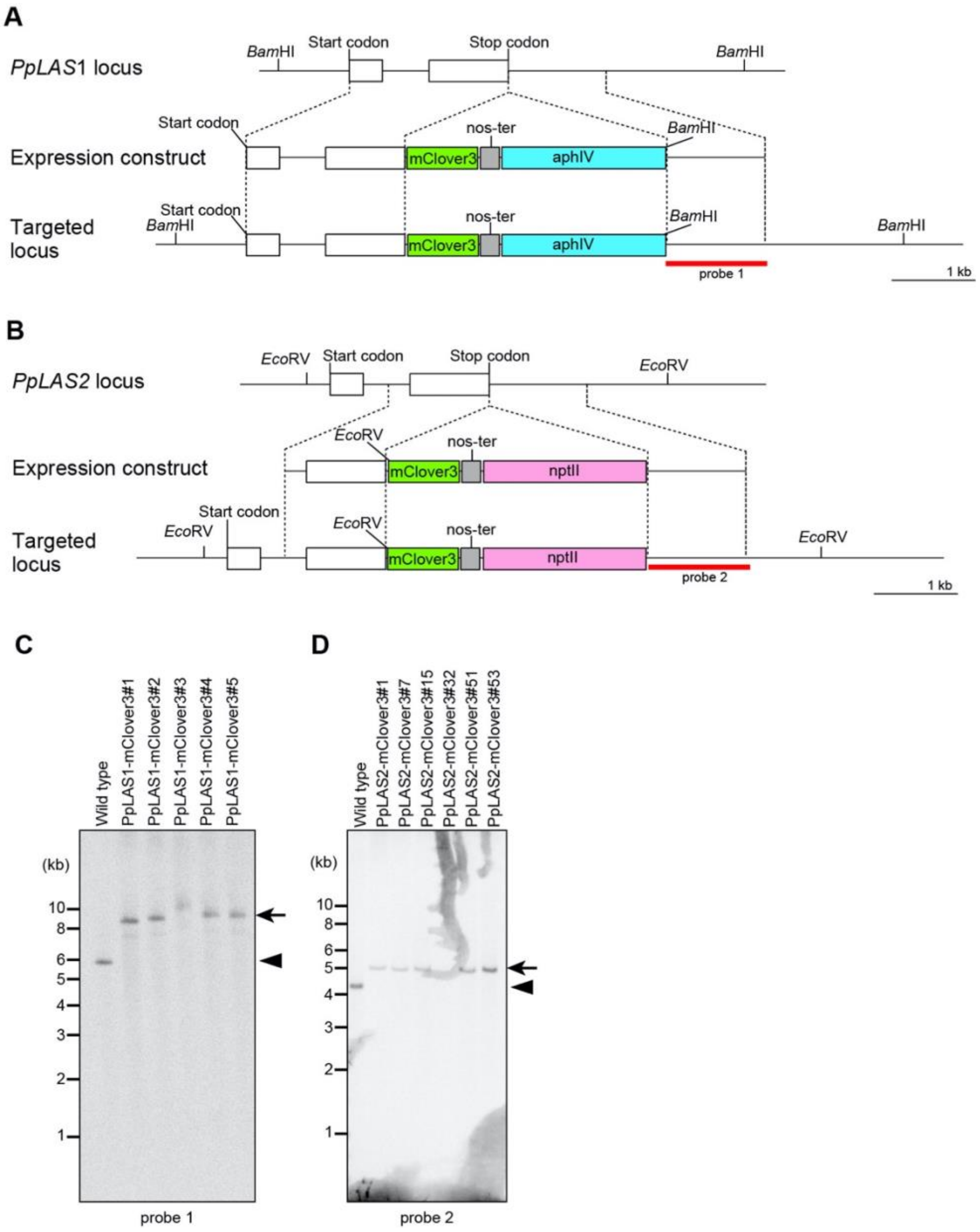

Fig. S15. Construction of PpLAS1-mClover3 and PpLAS2-mClover3 plants.
(A and B) Schematic diagrams of the strategy for the insertion of *mClover3* (28) at the 3' end of *PpLAS1* (A) or *PpLAS2* (B) coding regions. White boxes represent exons. Green, gray, orange and magenta boxes denote *mClover3* (green) (28), the nopaline synthase terminator (nos-ter: gray) (7), the aminoglycoside phosphotransferase IV expression cassette (aphIV: cyan) (8), and the neomycin phosphotransferase II expression cassette (nptII: pink) (7) are shown in different colors. Probes used in (C and D) are indicated as red horizontal lines. To insert *mClover3* in frame with the *PpLAS1* coding sequence, a genomic DNA fragment from the middle to the last codon was PCR amplified from wild-type genomic DNA. The amplified fragment was inserted into the 5' end of the coding region of *Citrine* in the pCit-aphIV plasmid (LC703380) in-frame. A genomic fragment containing the 3' flanking region of *PpLAS1* was inserted into the 3' region of the aphIV expression cassette. *Citrine* in the resulting plasmid was replaced with *mClover3* in the pmClo3-nptII plasmid (ON092627) to place *mClover3* in frame with the *PpAS1* coding sequence. To insert *mClover3* in frame with the *PpLAS2* coding sequence, a *PpLAS2* genomic DNA fragment extending from the middle to the last codon was PCR amplified from wild-type genomic DNA. The amplified fragment was inserted into the 5' end of the coding region of

mClover3 in the pmClo3-nptII plasmid in-frame. Genomic fragments containing the 3’ flanking region of *PpLAS2* were inserted into the 3’ region of the nptII expression cassettes of the resulting plasmids. The generated constructs were digested by suitable restriction enzymes for gene targeting.

(C) DNA gel blot analysis of PpLAS1-mClover3 lines. Genomic DNA of wild-type and PpLAS1-mClover3 (#1, #2, #3, #4, and #5) plants was digested with *Bam*HI*.* The arrowhead and arrow indicate DNA fragments specific to wild-type and PpLAS1-mClover3 plants, respectively.

(D) DNA gel blot analysis of PpLAS2-mClover3 lines. Genomic DNA of wild-type and PpLAS2-mClover3 (#1, #7, #15, #32, #51, and #53) plants was digested with *Hin*dIII. The arrowhead and arrow indicate DNA fragments specific to wild-type and PpLAS2-mClover3 plants, respectively.

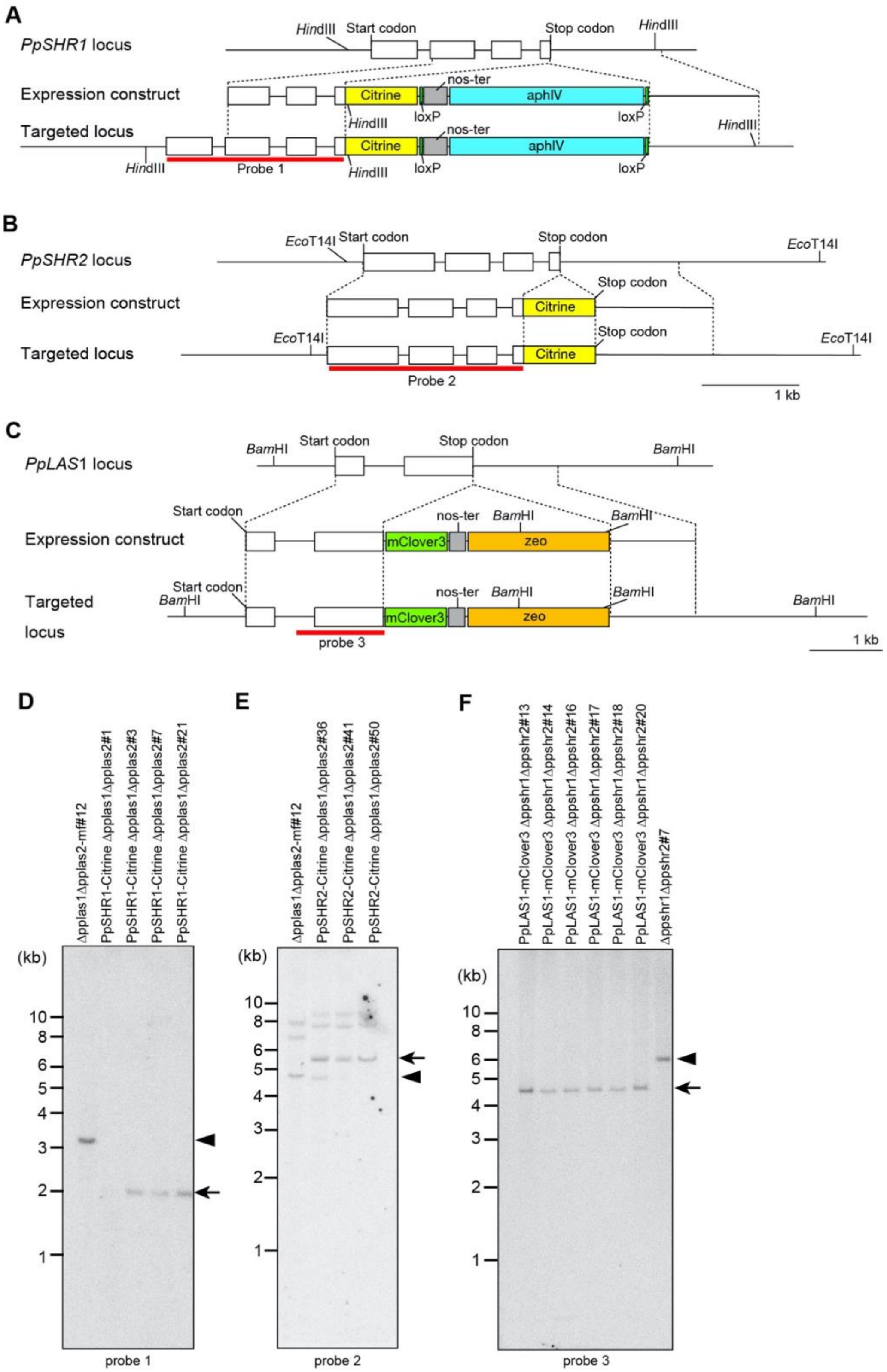
A
PpSHR1 locus
HindIII
Start codon
Stop codon
Expression construct
Citrine
nos-ter
aphIV
loxP
Targeted locus
Probe 1
B
PpSHR2 locus
EcoT14I
Probe 2
1 kb
C
PpLAS1 locus
BamHI
mClover3
zeo
probe 3
D
Δpplas1Δpplas2-mf#12
PpSHR1-Citrine Δpplas1Δpplas2#1
PpSHR1-Citrine Δpplas1Δpplas2#3
PpSHR1-Citrine Δpplas1Δpplas2#7
PpSHR1-Citrine Δpplas1Δpplas2#21
(kb)
probe 1
E
PpSHR2-Citrine Δpplas1Δpplas2#36
PpSHR2-Citrine Δpplas1Δpplas2#41
PpSHR2-Citrine Δpplas1Δpplas2#50
probe 2
F
PpLAS1-mClover3 Δppshr1Δppshr2#13
PpLAS1-mClover3 Δppshr1Δppshr2#14
PpLAS1-mClover3 Δppshr1Δppshr2#16
PpLAS1-mClover3 Δppshr1Δppshr2#17
PpLAS1-mClover3 Δppshr1Δppshr2#18
PpLAS1-mClover3 Δppshr1Δppshr2#20
Δppshr1Δppshr2#7
probe 3

Fig. S16. Construction of PpSHR1-CitrineΔpplas1Δpplas2, PpSHR2-CitrineΔpplas1Δpplas2, and PpLAS1-mClover3Δppshr1Δppshr2 plants.
(A and B) Schematic diagrams of the strategy used to insert *Citrine* at the 3' end of the endogenous *PpSHR1* (A) and *PpSHR2* (B) genes. White boxes represent exons. *Citrine* (yellow) (11), loxP (green) (9), the nopaline synthase terminator (nos-ter: gray) (7), and the aminoglycoside phosphotransferase IV expression cassette (aphIV: cyan) (8) are shown in different colors. Probes used in (D and E) are indicated as red horizontal lines. To insert *Citrine* at the 3' ends of *PpSHR1* (A) and *PpSHR2* (B), the PpSHR1-Citrine (Fig. S9*A*) and PpSHR2-Citrine (Fig. S3*C*) expression constructs were introduced into Δpplas1Δpplas2-mf#12 plants (Fig. S14), respectively.
(C) Schematic diagram of the strategy used to insert *mClover3* at the 3' end of the endogenous *PpLAS1* gene. White boxes represent exons. Green, gray, and orange boxes denote *mClover3* (28), the nopaline synthase terminator (nos-ter) (7), and the zeocin resistance cassette (zeo) (16), respectively. The probe used in (F) is indicated as a red horizontal line. The aphIV expression cassette in the PpLAS1-mClover*3* expression construct (Fig. S15*A*) was replaced with the zeocin resistance cassette. The generated construct was digested with suitable restriction enzymes for gene targeting and introduced into Δppshr1Δppshr2#7 plants.
(D) DNA gel-blot analysis of PpSHR1-CitrineΔpplas1Δpplas2 plants. Genomic DNA of Δpplas1Δpplas2-mf#12 and PpSHR1-CitrineΔpplas1Δpplas2 (#1, #3, #7, and #21) plants was digested with *Hin*dIII. The arrowhead and arrow indicate DNA fragments specific to Δpplas1Δpplas2-mf#12 and PpSHR1-CitrineΔpplas1Δpplas2 plants, respectively.
(E) DNA gel blot analysis of PpSHR2-CitrineΔpplas1Δpplas2 plants. Genomic DNA of Δpplas1Δpplas2-mf#12 and PpSHR2-CitrineΔpplas1Δpplas2 (#36, #41, and #50) plants was digested with *Eco*T14I. The arrowhead and arrow indicate DNA fragments specific to Δpplas1Δpplas2-mf#12 and PpSHR2-CitrineΔpplas1Δpplas2 plants, respectively.
(F) DNA gel blot analysis of PpLAS1-mClover3Δppshr1Δppshr2 plants. Genomic DNA of Δppshr1Δppshr2#7 and PpLAS1-mClover3Δppshr1Δppshr2 (#13, 14, #16, #17, #18, and #20) plants was digested with *Bam*HI. The arrowhead and arrow indicate DNA fragments specific to Δppshr1Δppshr2#7 and PpLAS1-mClover3Δppshr1Δppshr2 plants, respectively.

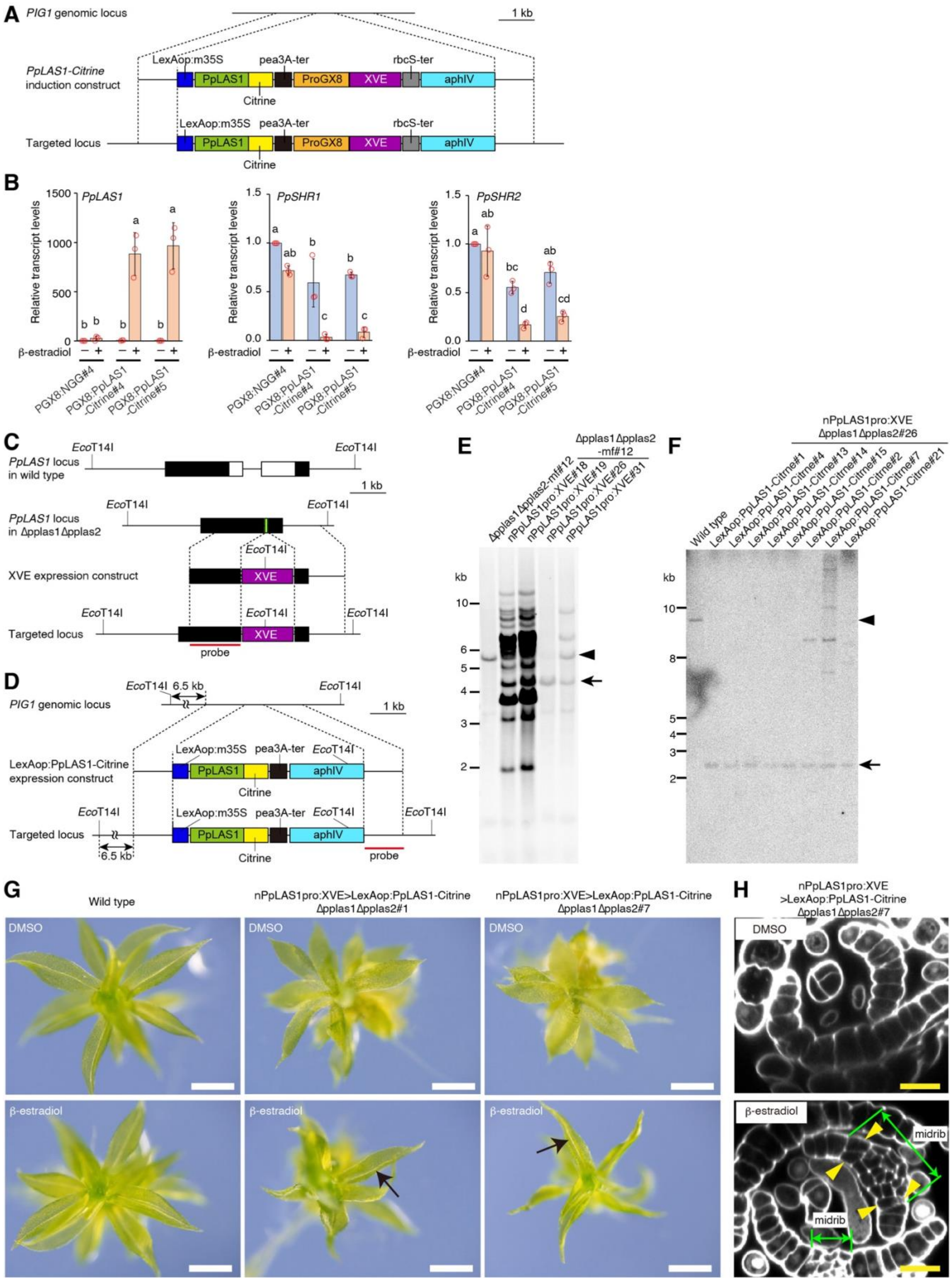

A
PIG1 genomic locus
1 kb
PpLAS1-Citrine induction construct
LexAop:m35S
PpLAS1
Citrine
pea3A-ter
ProGX8
XVE
rbcS-ter
aphIV
Targeted locus
B
PpLAS1
PpSHR1
PpSHR2
Relative transcript levels
β-estradiol
PGX8:NGG#4
PGX8:PpLAS1-Citrine#4
PGX8:PpLAS1-Citrine#5
C
EcoT14I
PpLAS1 locus in wild type
PpLAS1 locus in Δpplas1Δpplas2
XVE expression construct
probe
D
6.5 kb
LexAop:PpLAS1-Citrine expression construct
E
Δpplas1Δpplas2-mf#12
nPpLAS1pro:XVE#18
nPpLAS1pro:XVE#19
nPpLAS1pro:XVE#26
nPpLAS1pro:XVE#31
kb
F
nPpLAS1pro:XVE Δpplas1Δpplas2#26
Wild type
LexAop:PpLAS1-Citrne#1
LexAop:PpLAS1-Citrne#4
LexAop:PpLAS1-Citrne#13
LexAop:PpLAS1-Citrne#14
LexAop:PpLAS1-Citrne#15
LexAop:PpLAS1-Citrne#2
LexAop:PpLAS1-Citrne#7
LexAop:PpLAS1-Citrne#21
G
Wild type
nPpLAS1pro:XVE>LexAop:PpLAS1-Citrine Δpplas1Δpplas2#1
nPpLAS1pro:XVE>LexAop:PpLAS1-Citrine Δpplas1Δpplas2#7
DMSO
β-estradiol
H
nPpLAS1pro:XVE >LexAop:PpLAS1-Citrine Δpplas1Δpplas2#7
midrib

Fig. S17. Construction of GX8:PpLAS1-Citrine and nPpLAS1pro:XVE>LexAop:PpLAS1-CitrineΔpplas1Δpplas2 plants and PpLAS1-Citrine induction

(A) Schematic diagram showing the insertion of the *PpLAS1-Citrine* induction construct into the *PIG1* putative neutral genomic locus (12). The connected DNA fragment of the *LexA* operator and minimal *35S* promoter (blue: LexAop:m35S) (13), *PpLAS1* (green), *Citrine* (yellow) (11), the pea *rbcS3A* terminator (black: pea3A-ter) (14), the putative *ProGX8* promoter (orange) (12), a DNA fragment encoding an XVE fusion protein derived from pER8 (purple) (13), the *rbcS* terminator (rbcS-ter: gray) (14), and the aminoglycoside phosphotransferase IV expression cassette (aphIV： cyan) (8) are shown in different colors. To produce the *PpLAS1-Citrine* induction construct, the *PpLAS1*-coding sequence was inserted into the *Kpn*I and *Cla*I sites of the pCit-aphIV plasmid (LC703380), resulting in the generation of the in-frame *PpLAS1-Citrine* fusion. The *PpLAS1-Citrine* fragment was then PCR amplified and inserted into the pENTR/D-TOPO vector (ThermoFisher Scientific) to generate the pENTR:PpLAS1-Citrine plasmid. The pENTR:PpLAS1-Citrine plasmid was subjected to LR reaction using the destination vector pPGX8 (AB537482) (12) to generate the GX8:PpLAS1-Citrine plasmid. The generated construct was digested with the restriction enzyme *Pme*I for gene targeting and introduced into wild-type plants.

(B) Changes in the *PpSHR1* and *PpSHR2* transcripts after the Induction of *PpLAS1-Citrine* in gametophores of GX8:PpLAS1-Citrine plants. Gametophores of GX8:NLS-sGFP-GUS#4 (GX8:NGG#4) (12) and GX8:PpLAS1-Citrine (#4 and #5) plants cultivated for 3 weeks were soaked in liquid BCDAT medium with DMSO or 1 μM β-estradiol for three days. Total RNA was purified from gametophores for RT-qPCR analysis. Relative *PpLAS1*, *PpSHR1*, and *PpSHR2* transcript levels were obtained by normalization to that of *Pp3c9_17670* transcript encoding a thiosulfate sulfurtransferase (15); the value for each transcript in GX8:NGG#4 plants with DMSO was set to 1.0. The data points shown in red circles are the averages of three technical replicates in each plant, and the bars indicate the means of the relative transcript levels from three biological replicates. Error bars indicate standard deviations (SD). Lowercase letters indicate significant differences (one-way ANOVA and Tukey's test, $p < 0.05$).

(C) Schematic diagram showing the insertion of the *XVE* DNA fragment into the *PpLAS1* locus in Δpplas1Δpplas2 plant. A DNA fragment encoding an XVE fusion protein (purple) derived from pER8 (13) was introduced into the *PpLAS1* locus in Δpplas1Δpplas2-mf#12 plant (Fig. S14). The probe used in (C) is indicated as a red horizontal line. To generate the XVE induction construct, a DNA fragment encoding XVE, a genomic DNA fragment including a partial sequence of the 5' untranslated region of *PpLAS1*, a genomic fragment including the 3'-untranslated region of *PpLAS1*, and the pUC19 plasmid were individually PCR amplified and precisely assembled using the In-Fusion HD Cloning Kit (TAKARA BIO). The generated construct, nPpLAS1pro:XVE, was digested with the restriction enzyme *Eco*RV for gene targeting and introduced into Δpplas1Δpplas2-mf#12 plants to generate the nPpLAS1pro:XVEΔpplas1Δpplas2 plants. Note that the *XVE* expression is activated by the native *PpLAS1* promoter activity.

(D) Schematic diagram showing the insertion of the LexAop:PpLAS1-Citrine expression construct into the *PIG1* putative neutral genomic locus in the nPpLAS1pro:XVEΔpplas1Δpplas2#26 plant. A connected DNA fragment of *LexAop:m35S* (blue), *PpLAS1* (green), *Citrine* (yellow) (11), pea3A-ter (black), and the aphIV expression cassette (cyan) (8) are shown in different colors. To produce the LexAop:PpLAS1-Citrine expression construct, the GX8:PpLAS1-Citrine plasmid was digested with the restriction enzyme *Eco*T22I and then self-ligated to remove the putative *ProGX8* promoter, a DNA fragment encoding the XVE fusion protein, and the *rbcS* terminator. In response to β-estradiol, PpLAS1-Citrine is overexpressed under the control of the native *PpLAS1* promoter activity.

(E) DNA gel-blot analysis of nPpLAS1pro:XVEΔpplas1Δpplas2 plants. Genomic DNA of Δpplas1Δpplas2-mf#12 and nPpLAS1pro:XVEΔpplas1Δpplas2 (#18, #19, #26, and #31) plants was digested with *Eco*T14I. The arrowhead and arrow indicate DNA fragments specific to Δpplas1Δpplas2-mf#12 and nPpLAS1pro:XVEΔpplas1Δpplas2 plants, respectively.

(F) DNA gel blot analysis of nPpLAS1pro:XVE>LexAop:PpLAS1-CitrineΔpplas1Δpplas2 plants. Genomic DNA of wild type and nPpLAS1pro:XVE> LexAop:PpLAS1-CitrineΔpplas1Δpplas2 (#1, #2, #4, #7, #13, #14, #15, and #21) plants was digested with *Eco*T14I. The arrowhead and arrow indicate DNA fragments specific to wild type and nPpLAS1pro:XVE> LexAop:PpLAS1-CitrineΔpplas1Δpplas2 plants, respectively.

(G) Induction of *PpLAS1-Citrine* in nPpLAS1pro:XVE> LexAop:PpLAS1-CitrineΔpplas1Δpplas2 plants. Wild type and nPpLAS1pro:XVE> LexAop:PpLAS1-CitrineΔpplas1Δpplas2 (#1 and #7) plants were cultivated on solid BCDAT medium with DMSO or 1 μM β-estradiol for one month. Black arrows indicate midrib. (Scale bar, 500 μm.)

(H) Representative optical transverse sections of young leaves in nPpLAS1pro:XVE>PpLAS1-CitrineΔpplas1Δpplas2 plants treated with DMSO or β-estradiol. Gametophores of nPpLAS1pro:XVE> LexAop:PpLAS1-CitrineΔpplas1Δpplas2#7 plants were cultivated in the presence of 1 μM β-estradiol or DMSO for

4 days, fixed in 4% (w/v) paraformaldehyde, stained with calcofluor white, and then cleared with TOMEI solution (29). Gametophores of nPpLAS1pro:XVE> LexAop:PpLAS1-CitrineΔpplas1Δpplas2#7 plants with β-estradiol formed midrib in leaves. Yellow arrowheads indicate daughter cells of the periclinally divided mml cells. (Scale bar, 20 µm.)

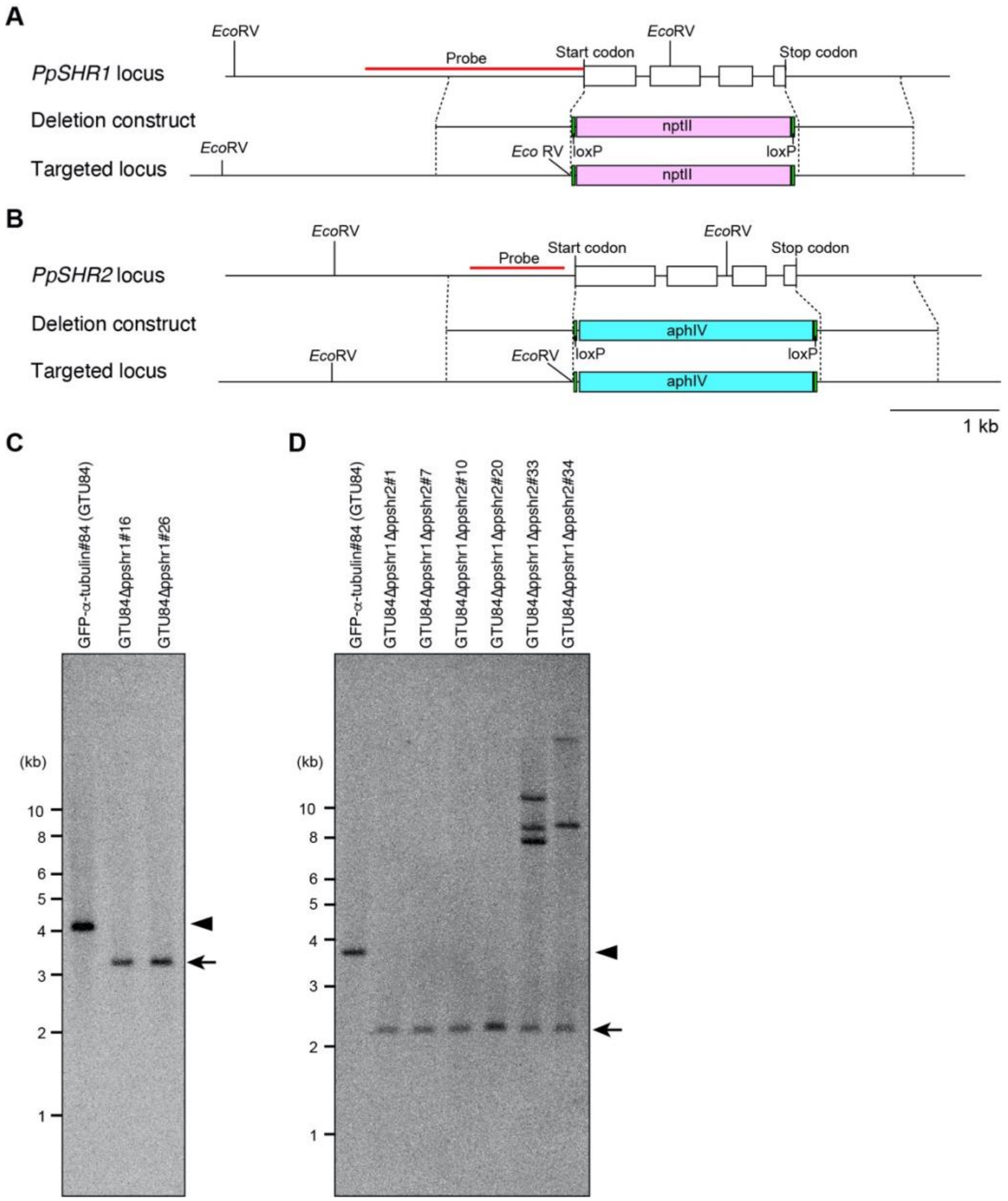

Fig. S18. Construction of the GFP-α-TubulinΔppshr1Δppshr2 deletion mutant plants.
(A and B) Schematic diagrams of the construct targeting the *PpSHR1* (A) and *PpSHR2* (B) loci. White boxes represent exons. Magenta and cyan boxes denote the neomycin phosphotransferase II expression cassette (nptII) (7) and aminoglycoside phosphotransferase IV expression cassette (aphIV) (8), respectively. Green boxes denote loxP sequences (9). Probes used in (C and D) are indicated as red horizontal lines. To visualize microtubules, the GFP-α-Tubulin expression construct (8) was introduced into wild-type plants to generate the GFP-α-Tubulin#84 (GTU84) line. The *PpSHR1* gene deletion construct (Fig. S2*A*) was introduced into the GTU84 plant to generate GTU84Δppshr1Δppshr2 plants (C). The GTU84Δppshr1#16 plant was used to generate the GTU84Δppshr1Δppshr2 double deletion mutants.
(C and D) DNA gel blot analyses of targeted plants with the probes indicated in (A and B). Genomic DNA of GTU84, GTU84Δppshr1 (#16 and #26), and GTU84Δppshr1Δppshr2 (#1, #7, #10, #20, #33 and #34) was digested with *Eco*RV. Arrowheads and arrows indicate DNA fragments specific to GTU84 and GTU84Δppshr1 or

GTU84Δppshr1 Δppshr2 plants, respectively. GTU84Δppshr1Δppshr2#1 was used for further analysis (Fig. 4 and Fig. S19).

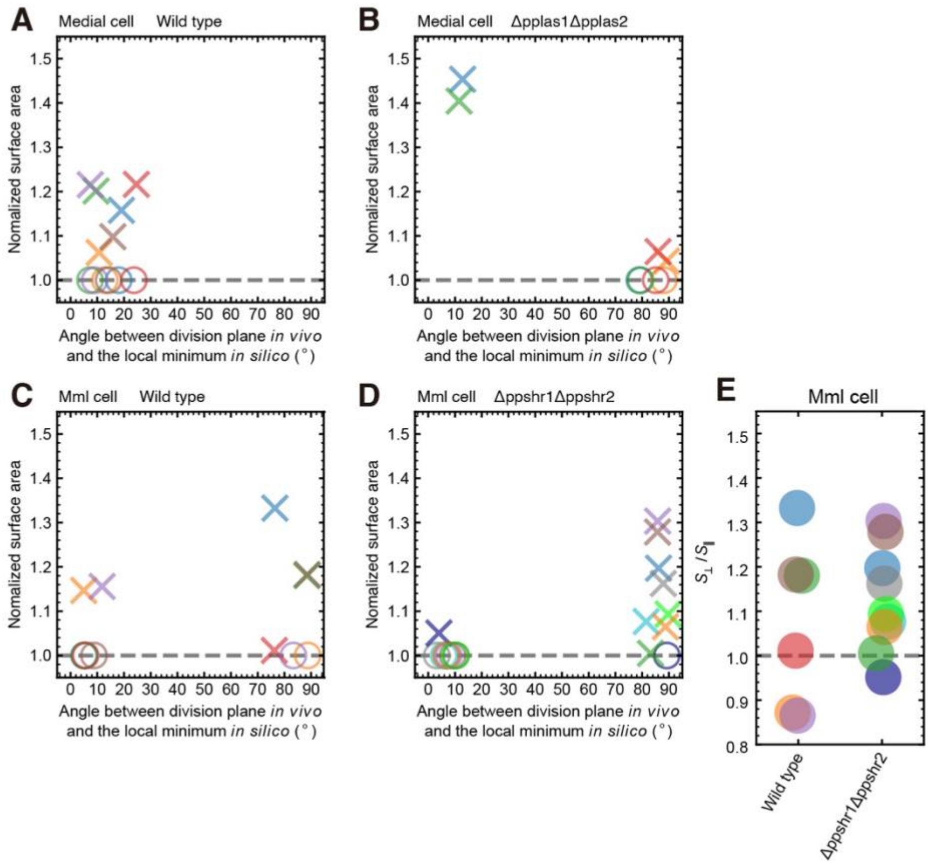

Fig. S19. The surface area of the division planes in the phase-field model simulations.
(A-D) The surface area (Eq. 2-7) of the non-global local minimal plane (cross) normalized by that of the global minimal plane (circle) is plotted as a function of the angular difference from the observed division plane orientation (Eq. 9). (E) The area of the local minimal plane orthogonal to the observed division plane ($S_{\perp}$, i.e., at the angular difference ~ 90 degrees in C, D) was divided by the area of the plane parallel to the observed plane ($S_{\parallel}$, at the angular difference ~ 0 degrees in C, D). In A-E, the colors of marks correspond to the examined cells in Fig. 4. Wild-type or GTU84 (*A*) shown in Fig. S18, ∆pplas1∆pplas2 (*B*), GTU84 (*C, E*), and GTU84∆ppshr1∆ppshr2 (*D, E*) plants were observed.

Table S1. Primer sequences used for plasmid constructions.

Construct	Orientation	Sequence
PpSHR1		
Δppshr1	5’ (F)	5'-CCATCGATGCTCAATGCAATGCTAGTGCAGCTG-3'
	5’ (R)	5'-CGGATATCGGGCTACCGTTCTAAGGTATTCAC-3'
	3’ (F)	5'-TCCCCCGGGTGAATCATCCAGATCTCTCAAC-3'
	3’ (R)	5'-GGCTCCTCGCGGCCGCTAACCTACAAATCTTCAG-3'
PpSHR1-mCitrine	5’ (F)	5'-CGACGGTATCGATAAGCTGCGGCCGCGATATATCTTCTGACGCGATGCACATC-3'
	5’ (R)	5'-CACCAAGATATCAAGCTTGCTTCCTCCTCCTCCATATGACGGGGTTGTGATGAGCTTCC-3'
	3’ (F)	5'-CCACGTGCCCGGGACTAGTGAATCATCCAGATCTCTCAACC-3'
	3’ (R)	5'-CGGCCGCTCTAGAACTAGCAGCACTTTGACAAAGCTTGCTCG-3'
PpSHR1-Citrine	5’ (F)	5'-CTCCTCGTCGAGACGGAGCTCTTCTGGAGACGATG-3'
	5’ (R)	5'-CCATCGATATATGACGGGGTTGTGATGAGCTTC-3'
	3’ (F)	5'-GACTAGTTGAATCATCCAGATCTCTCAACC-3'
	3’ (R)	5'-GGCTCCTCGCGGCCGCTAACCTACAAATCTTCAG-3'
PpSHR1-3×Myc	5’ (F)	5'-GGGTCGACTGCGAATCCTGTCAAGCCTTCATTC-3'
	5’ (R)	5'-GCCGCCGCCATATGACGGGGTTGTGATGAGCTTC-3'
	3’ (F)	5'-ATCATCCAGATCTCTCAACCTTTAAAAAGTATCATAAAATAG-3'
	3’ (R)	5'-GGGCGCCGCGACAAACATTCTTTCCCCGGAGGAC-3'
pPpU6-*PpSHR1*-sgRNA	(F)	5'-CATGAAGAAGTAGGGGTTACCATC-3'
	(R)	5'-AAACGATGGTAACCCCTACTTCTT-3'
PpSHR2		
Δppshr2	5’ (F)	5'-CCATCGATTGGTTGCTCCAGTTGAGTCACCG-3'
	5’ (R)	5'-CGGATATCGACCTGGATCACGACGAACTG-3'
	3’ (F)	5'-TCCCCCGGGTGATCTAAGATGAGTAGCCGTG-3'
	3’ (R)	5'-GGCTCCTCGCGGCCGCTTTAGATTTAAGACAACAC-3'
PpSHR2-mCitrine	5’ (F)	5'-CGACGGTATCGATAAGCTGCGGCCGCCGTCGCACCAATGCATCTAGTTTC-3'
	5’ (R)	5'-CACCAAGATATCAAGCTTGCTTCCTCCTCCTCCTGGTTCCGGTGACGGGCTTG-3'
	3’ (F)	5'-CCACGTGCCCGGGACTAGTGATCTAAGATGAGTAGCCGTGGCTAG-3'
	3’ (R)	5'-CGGCCGCTCTAGAACTAGCACCAAAAAAGGTTAAAATATCTTATTTATTGGC-3'
PpSHR2-Citrine	Vector (F)	5'-AGATCTTTAAGCTTGAGTATTCTATAGTGTCA-3'
	Vector (R)	5'-TTGTCCATGGTACCGAGCTCGAATTCGCCCTA-3'
	gORF (F)	5'-TCGGTACCATGGACAAACTTCTGGTAAAATAT-3'
	gORF (R)	5'-ATATCAAGTGGTTCCGGTGACGGGCTTGCGAT-3'
	Citrine (F)	5'-CGGAACCACTTGATATCTTGGTGAGCAAGGGC-3'
	Citrine (R)	5'-ATCTTAGATTACTTGTACAGCTCGTCCATGCC-3'
	3’ (F)	5'-ACAAGTAATCTAAGATGAGTAGCCGTGGCTAG-3'
	3’ (R)	5'-TCAAGCTTAAAGATCTATTCTATATTATACAT-3'
PpSHR2-3×Myc	5’ (F)	5'-GGCTCGAGCGGGCATTGAACATTTCAACCCG-3'
	5’ (R)	5'-GCCGCCGCCTGGTTCCGGTGACGGGCTTG-3'
	3’ (F)	5'-TCTAAGATGAGTAGCCGTGGCTAGATCA-3'
	3’ (R)	5'-GGGGCGCCGCGCATGCGTAGCAGTAACGAATCC-3'
pENTR:PpSHR2cDNA-Citrine	Vector (F)	5'-AAGGGTGGGCGCGCCGACCCAGCTTTCTTG-3'
	Vector (R)	5'-AAGGGGGCGGCCGCGGAGCCTGCTTTTTTG-3'
	PpSHR2 (F)	5'-CCGCGGCCGCCCCCTTATGGACAAACTTCTGGTA-3'
	PpSHR2 (R)	5'-ATATCAAGTGGTTCCGGTGACGGGCTTGCGAT-3'
	Citrine (F)	5'-CGGAACCACTTGATATCTTGGTGAGCAAGGGC-3'
	Citrine (R)	5'-CGGCGCGCCCACCCTTTTACTTGTACAGCTCGTC-3'

Table S1 (continued). Primer sequences used for plasmid constructions.

Construct	Orientation	Sequence
pSHR2pro:PsSHR2	(F)	5'-GCCGCCCCCTTCACCATGGACAAACTTCTGGTAAAATATCAAGAG-3'
	(R)	5'-GGCGCGCCCACCCTTTGGTTCCGGTGACGGGCTTG-3'
pSHR2pro:NLS-eGFP-GUS	(F)	5'-TAGAGGATCCCCCCCCCCGTTAAAAATCATCAGATTATGGTAGTGC-3'
	(R)	5'-TTTTGGATCAGCCATCCCGACCTGGATCACGACGAACTG-3'
pPpU6-*PpSHR2*-sgRNA	(F)	5'-CATGTCCTCAAACCGACCAAATCG-3'
	(R)	5'-AAACCGATTTGGTCGGTTTGAGGA-3'
PpSCR1		
∆ppscr1-nptII	5’ (F)	5'-CGGGTACCTCTTCCTGGTCGAGTTACGATTCTAC-3'
	5’ (R)	5'-AAGGGCCCTTATCTGGTCTGACGTGGTGATGG-3'
	3’ (F)	5'-TGCGGCCGCAAGATATTAAACCTACCTGCTGG-3'
	3’ (R)	5'-TCCCCGCGGATAGTTCTACAATCAATCCTTCG-3'
∆ppcr1-zeo	5’ (F)	5'-GGGGTACCCGAATTTCGTGACGTGAGCCCTAC-3'
	5’ (R)	5'-AAGGGCCCACTCACCGTATTGCCTGCTCTC-3'
	3’ (F)	5'-TGCGGCCGCAAGATATTAAACCTACCTGCTGG-3'
	3’ (R)	5'-TCCCCGCGGATAGTTCTACAATCAATCCTTCG-3'
Citrine-PpSCR1	CDS (F)	5'-TTTGTACAGGTGGTATCATGGCTTTGGTATGTCCTAATCCAAGAG-3'
	CDS (R)	5'-CGTGTACACTATTCGTTTGTCCAAGCAGAAGCAGTG-3'
	promoter (F)	5'-GAAGGAGCGTCAAGTTGGAATATGG-3'
	promoter (R)	5'-AAGGGCCCTTATCTGGTCTGACGTGGTGATGG-3'
pENTR:Citrine-PpSCR1	(F)	5'-CACCATGGTGAGCAAGGGCGAGGAGCTGTTC-3'
	(R)	5'-CTATTCGTTTGTCCAAGCAGAAGCAGTGAAC-3'
PpLAS1		
∆pplas1	5’ (F)	5'-AAAGGTACCCACCCGAAGCATGGAGTTC-3'
	5’ (R)	5'-TTTGAATTCGAAGAGACAACTGCGGTCTGC-3'
	3’ (F)	5'-AAAGCGGCCGCCTTCAGTTCAATCGACTGGCAG-3'
	3’ (R)	5'-TTTGAGCTCTGAATGCATTTGAAGCCAGG-3'
PpLAS1-mClover3	5’ (F)	5'-CGGGGTACCATGCAATTGTTTGTGCCACCCTTG-3'
	5’ (R)	5'-CCATCGATGTTACTTGGATGCCAAGAGGAGAC-3'
	3’ (F)	5'-TTTGAGCTCTGAATGCATTTGAAGCCAGG-3'
	3’ (R)	5'-AAAGCGGCCGCCTTCAGTTCAATCGACTGGCAG-3'
PpLAS1-mClover3	(F)	5'-CGGGGTACCATGCAATTGTTTGTGCCACCCTTG -3'
	(R)	5'-CCATCGATGTTACTTGGATGCCAAGAGGAGAC-3'
pENTR:PpLAS1-Citrine	(F)	5'-CACCATGCAATTGTTTGTGCCACCCTTG-3'
	(R)	5'-GCAGGAATTCTTACTTGTACAGCTCGTC-3'
nPpLAS1pro:XVE	XVE (F)	5'-TTCATGAAATGAAAGCGTTAACGGC -3'
	XVE (R)	5'-CTAGAGACTCAGACTGTGGCAGG-3'
	5'-UTR (F)	5'-TAGAGGATATCATTGATGCGTTTAGATAAA-3'
	5'-UTR (R)	5'-GCTTTCATTTCATGAAGGCCGCC-3'
	3'-UTR (F)	5'-CAGTCTGAGTCTCTAGTATTGACCTTCA-3'
	3'-UTR (R)	5'-CCGGGGATATCATTATCTAAGGTACTGGA-3'
	pUC19 (F)	5'-ATAATGATATCCCCGGGTACCGA-3'
	pUC19 (R)	5'-TCAATGATATCCTCTAGAGTCGACC-3'

Table S1 (continued). Primer sequences used for plasmid constructions.

Construct	Orientation	Sequence
PpLAS2		
Δpplas2	5’ (F)	5'-AAAGGTACCATCGGCGCAAGTGAGTCAAG-3'
	5’ (R)	5'-TTTAAGCTTGTTTGACGAGGGCTGGAGAC-3'
	3’ (F)	5'-AAAGCGGCCGCTAGCGTAGTGTAGAAACGCCTGG-3'
	3’ (R)	5'-TTTGAGCTCCCCAGACGCATGGTATCTCG-3'
PpLAS2-mClover3	5’ (F)	5'-CCCCTCGAGTTAACACAATAATCATCATCTGGCAAC-3'
	5’ (R)	5'-TTATCGATCATGTTATTCGTATTCCAAGAAGAGACAC-3'
	3’ (F)	5'-GGGGATCCGCCACTCATATCGACATGAAGTTCG-3'
	3’ (R)	5'-GCTCTAGAGGCCTTGATCTGCCAGTAC-3'

(F), forward primer; (R), reverse primer

Table S2. Primer sequences used for genomic PCR to evaluate the *PpSHR2* locus

Primer position	Orientation	Sequence
1	F	5'-ATCTCTGATGAGACGATGCAGACTG-3'
	R	5'-AAGACTCGGTGGTCTGAGATGTG-3'
2	F	5'-GGTCGTTCGCTAGCAACACTGTAG-3'
	R	5'-CTGGATCACGACGAACTGGAAATGC-3'
3	F	5'-CATGATCTAAGATGAGTAGCCGTG-3'
	R	5'-GTGGAAGAAGTTTTAGATTTAAGACAACAC-3'
4	F	5'-CATATATCAGCAGGTCACACAAAAGCTAC-3'
	R	5'-AAACCGAACCAATCCGTGATTACCTGTG-3'
5	F	5'-GGTCGTTCGCTAGCAACACTGTAG-3'
	R	5'-GTGGAAGAAGTTTTAGATTTAAGACAACAC-3'
6	F	5'-CTAGCCTCTAATCGTCTTTTTACTC-3'
	R	5'-ATATTCGATGAACACAGACC-3'

F, forward primer; R, reverse primer

Table S3. Primer sequences used for RT-qPCR

Gene name	Orientation	Sequence
PpSHR1	F	5'-CGTCTCATGCTTGAGCGAAA-3'
	R	5'-CGCGCGTTCAAAGCATT-3'
PpSHR2	F	5'-CCAAGACAACTCCATGATCAAAGT-3'
	R	5'-CACCGGGCTCGCGTAGTAT-3'
PpSCR1	F	5'-TGCCAATCAGGCCATCTTC-3'
	R	5'-TCGATGACATGCACGTTGAA-3'
PpSCR2	F	5'-CCCTTTCGGCACGTCTGT-3'
	R	5'-CGAGACCCCATGCTTTCG-3'
PpSCR3	F	5'-CTCGGTGGAGACCCTCGAT-3'
	R	5'-CCCAAGGTTGCTGCGAAAT-3'
Pp3c9_17670	F	5'-CAACGGGAGCTTTCTCTAGCA-3'
	R	5'-GCGCTGTTTATTGTTTTCAACATC-3'

F, forward primer; R, reverse primer

Table S4. Primer sequences used for yeast two-hybrid assay

Gene name	Orientation	Sequence
PpSHR1	F	5'-GGGGACAAGTTTGTACAAAAAAGCAGGCTTGACTAATATCATGGCAC-3'
	R	5'-GGGGACCACTTTGTACAAGAAAGCTGGGTCTCAGTTTCATGAGCTCAC-3'
PpSHR2	F	5'-GGGGACAAGTTTGTACAAAAAAGCAGGCTTCGTGATCCAGGTCATGGAC-3'
	R	5'-GGGGACCACTTTGTACAAGAAAGCTGGGTGGCTACTCATCTTAGATCA-3'
PpSCR1	F	5'-GGGGACAAGTTTGTACAAAAAAGCAGGC TTGAGTATGGCTTTGGTATGT-3'
	R	5'-GGGGACCACTTTGTACAAGAAAGCTGGGTTCTCTTCGTTTGTCCAAGCAG-3'

F, forward primer; R, reverse primer

SI References

1. S. Q. Le, O. Gascuel, An improved general amino acid replacement matrix. *Mol. Biol. Evol.* **25**, 1307-1320 (2008).

2. S. F. Altschul *et al.*, Gapped BLAST and PSI-BLAST: a new generation of protein database search programs. *Nucleic Acids Res.* **25**, 3389-3402 (1997).

3. K. Katoh, D. M. Standley, MAFFT multiple sequence alignment software version 7: improvements in performance and usability. *Mol. Biol. Evol.* **30**, 772-780 (2013).

4. W. Maddison, D. Maddison, MESQUITE: a modular system for evolutionary analysis. Version 3.61. Available at http://www.mesquiteproject.org. Deposited 26 December 2019.

5. A. Stamatakis, RAxML version 8: a tool for phylogenetic analysis and post-analysis of large phylogenies. *Bioinformatics* **30**, 1312-1313 (2014).

6. J. Felsenstein, PHYLIP (Phylogeny Inference Package) version 3.697. Available at https://evolution.genetics.washington.edu/phylip.html. Deposited December 2017.

7. T. Nishiyama, Y. Hiwatashi, I. Sakakibara, M. Kato, M. Hasebe, Tagged mutagenesis and gene-trap in the moss, *Physcomitrella patens* by shuttle mutagenesis. *DNA Res.* **7**, 9-17 (2000).

8. Y. Hiwatashi *et al.*, Kinesins are indispensable for interdigitation of phragmoplast microtubules in the moss *Physcomitrella patens*. *Plant Cell* **20**, 3094-3106 (2008).

9. J. Odell, P. Caimi, B. Sauer, S. Russell, Site-directed recombination in the genome of transgenic tobacco. *Mol. Gen. Genet.* **223**, 369-378 (1990).

10. D. A. Zacharias, J. D. Violin, A. C. Newton, R. Y. Tsien, Partitioning of lipid-modified monomeric GFPs into membrane microdomains of live cells. *Science* **296**, 913-916 (2002).

11. A. A. Heikal, S. T. Hess, G. S. Baird, R. Y. Tsien, W. W. Webb, Molecular spectroscopy and dynamics of intrinsically fluorescent proteins: coral red (dsRed) and yellow (Citrine). *Proc. Natl. Acad. Sci. U.S.A.* **97**, 11996-12001 (2000).

12. M. Kubo *et al.*, System for stable beta-estradiol-inducible gene expression in the moss *Physcomitrella patens*. *PLoS One* **8**, e77356 (2013).

13. J. Zuo, Q. W. Niu, N. H. Chua, Technical advance: An estrogen receptor-based transactivator XVE mediates highly inducible gene expression in transgenic plants. *Plant J.* **24**, 265-273 (2000).

14. R. Fluhr, P. Moses, G. Morelli, G. Coruzzi, N. H. Chua, Expression dynamics of the pea rbcS multigene family and organ distribution of the transcripts. *EMBO J.* **5**, 2063-2071 (1986).

15. M. Ishikawa *et al.*, Physcomitrella STEMIN transcription factor induces stem cell formation with epigenetic reprogramming. *Nat. Plants* **5**, 681-690 (2019).

16. K. Sakakibara, T. Nishiyama, H. Deguchi, M. Hasebe, Class 1 KNOX genes are not involved in shoot development in the moss *Physcomitrella patens* but do function in sporophyte development. *Evol. Dev.* **10**, 555-566 (2008).

17. R. A. Jefferson, T. A. Kavanagh, M. W. Bevan, GUS fusions: b-glucuronidase as a sensitive and versatile gene fusion marker in higher plants. *EMBO J.* **6**, 3901-3907 (1987).

18. D. Kalderon, W. D. Richardson, A. F. Markham, A. E. Smith, Sequence requirements for nuclear location of simian virus 40 large-T antigen. *Nature* **311**, 33-38 (1984).

19. K. Tamura, M. Kimura, I. Yamaguchi, Blasticidin S deaminase gene (BSD): a new selection marker gene for transformation of *Arabidopsis thaliana* and *Nicotiana tabacum*. *Biosci. Biotechnol. Biochem.* **59**, 2336-2338 (1995).

20. M. Ishikawa *et al.*, Physcomitrella cyclin-dependent kinase A links cell cycle reactivation to other cellular changes during reprogramming of leaf cells. *Plant Cell* **23**, 2924-2938 (2011).

21. D. Kurihara, Y. Mizuta, Y. Sato, T. Higashiyama, ClearSee: a rapid optical clearing reagent for whole-plant fluorescence imaging. *Development* **142**, 4168-4179 (2015).

22. D. T. Jones, W. R. Taylor, J. M. Thornton, The rapid generation of mutation data matrices from protein sequences. *Comput. Appl. Biosci.* **8**, 275-282 (1992).

23. J. E. DiCarlo *et al.*, Genome engineering in Saccharomyces cerevisiae using CRISPR-Cas systems. *Nucleic Acids Res.* **41**, 4336-4343 (2013).

24. C. Collonnier *et al.*, CRISPR-Cas9-mediated efficient directed mutagenesis and RAD51-dependent and RAD51-independent gene targeting in the moss *Physcomitrella patens*. *Plant Biotechnol. J.* **15**, 122-131 (2017).

25. J. Zhang *et al.*, The hornwort genome and early land plant evolution. *Nat. Plants* **6**, 107-118 (2020).

26. F. W. Li *et al.*, Anthoceros genomes illuminate the origin of land plants and the unique biology of hornworts. *Nat. Plants* **6**, 259-272 (2020).

27. S. Cheng *et al.*, Genomes of subaerial Zygnematophyceae provide insights into land plant evolution. *Cell* **179**, 1057-1067 (2019).

28. B. T. Bajar *et al.*, Improving brightness and photostability of green and red fluorescent proteins for live cell imaging and FRET reporting. *Sci. Rep.* **6**, 20889 (2016).

29. J. Hasegawa *et al.*, Three-Dimensional Imaging of Plant Organs Using a Simple and Rapid Transparency Technique. *Plant Cell Physiol* **57**, 462-472 (2016).
